# A disintegrin and metalloproteinase with thrombospondin motifs 18 (ADAMTS18) cleaves fibronectin and negatively regulates its fibrillogenesis

**DOI:** 10.1016/j.jbc.2025.110844

**Published:** 2025-10-22

**Authors:** Maria Barbiera, Mikko Gynther, Tetsuya Terasaki, Suvi Jauhiainen, Johanna P. Laakkonen, Michael Jeltsch, Seppo Ylä-Herttuala, Nihay Laham-Karam

**Affiliations:** 1 A. I. Virtanen Institute for Molecular Sciences, University of Eastern Finland, Kuopio, Finland; 2 School of Pharmacy, University of Eastern Finland, Kuopio, Finland; 3 Division of Pharmaceutical Biosciences, Faculty of Pharmacy, University of Helsinki, Helsinki, Finland; 4 Heart Center, Kuopio University Hospital, Kuopio, Finland

**Keywords:** ADAMTS18, angiogenesis, fibronectin, endothelial cell, endothelial sprouting, extracellular matrix remodeling, fibrillogenesis, lentiviral vector, mass spectrometry, siRNA

## Abstract

Remodeling of the extracellular matrix (ECM) plays a crucial role in the development, maintenance, and repair of all tissues. Therefore, identifying the regulators of this process is essential. Among these, A disintegrin and metalloproteinase with thrombospondin motifs 18 (ADAMTS18) has been implicated in fibronectin (FN) matrix regulation. Knockout of *ADAMTS18*, either in mouse models or *in vitro*, was shown to lead to FN accumulation, mutation in epithelial branching, and reduction in endothelial sprouting. However, the mechanisms by which ADAMTS18 influences endothelial-specific functions and the ECM, particularly in the regulation of FN fibrils, remain unclear. In this study, using both siRNA-mediated knockdown and overexpression of ADAMTS18 in primary endothelial cells (ECs), we delineated some of these mechanisms. Using global RNA-Seq of ECs, we demonstrated differential gene regulation of vessel development and endothelial adhesion genes with ADAMTS18 siRNA knockdown, whereas cell matrix– and cell cycle–associated genes were affected by overexpression of ADAMTS18. Consistent with the latter, we observed reduced EC proliferation and altered cell cycle with ADAMTS18 overexpression. Using mass spectrometry, we identified two sites in FN that are proteolytically cleaved by ADAMTS18, including a cleavage site in the linker FN-I_5-6_. Cleavage at this site generated FN molecules lacking the N-terminal FN-I_1–5_ (29 kDa) fragment that is known to be essential for FN fibrillogenesis. Accordingly, ADAMTS18 overexpression greatly impaired FN fibrillogenesis in endothelial cultures and in coculture with fibroblasts. Our results implicate ADAMTS18 in FN-associated ECM remodeling and suggest an important role for ADAMTS18 in endothelium biology.

A disintegrin and metalloproteinase with thrombospondin motifs (ADAMTS) family of zinc metalloproteinases (1) includes 19 enzymes that are proteolytically active in a variety of physiological processes and have been linked to the pathogenesis of different diseases, such as vascular pathologies, arthritis, bronchopulmonary dysplasia, and cancer (2, 3). Some of the ADAMTS family members have been well characterized, such as ADAMTS13, which regulates Von Willebrand factor in homeostasis (4), and ADAMTS3, which plays an essential role in processing of vascular endothelial growth factor C (VEGF-C), a factor critical for lymphangiogenesis (5). Other ADAMTSs participate in the remodeling of the extracellular matrix (ECM), regulating the cleavage of aggrecan and proteoglycans (ADAMTS1, 4, 5, 8, 9, 15, and 20), collagen (ADAMTS2, 3, and 14), and fibronectin (FN) (6, 7). However, some members of the family, such as ADAMTS18, remain with the “orphan” designation, because of insufficient knowledge of their substrate, which also limits understanding of their mechanisms. First identified in 2002 (8), ADAMTS18 has been associated with different diseases, such as cancer and bone- and eye-related disorders (9, 10). In mouse knockout models, ADAMTS18 was observed to affect the development of the lens, lung, female reproductive tract (11) and kidney (12). Our group recently suggested an uncharacterized function of ADAMTS18 in endothelial cells (ECs), as *ADAMTS18* expression was found to be regulated by the endothelial-specific superenhancer SE12313 in primary ECs, and siRNA knockdown of *ADAMTS18* mRNA had a detrimental effect on endothelial sprout formation *in vitro* (13). Of note, *Adamts18* deficiency *in vivo* induced aberrant EC behavior in the intestinal villi and in the liver sinusoids, because of dysregulation of the vasculogenic factor, VEGF-A distribution surrounding ECs (14, 15). Interestingly, this alteration in VEGF-A distribution was associated with FN accumulation (14, 15). In *Adamts18* knockout mice, FN accumulation was also observed in the pubertal mammary glands (16), the common carotid artery (17), and the mesenchyme of embryonic lacrimal glands (18).

FN is one of the major components of the ECM. It is a secreted dimeric glycoprotein with a monomer size spanning from 230 to 270 kDa depending on alternative splicing (19, 20). Each monomer consists of three structurally different repeating module types I to III. Once secreted, FN dimers undergo fibrillogenesis, which is a cell-mediated process leading to fiber formation, which relies on the initial binding of FN to cellular integrins through Arg-Gly-Asp (RGD) cell-binding sites (21, 22). Binding to integrins induces a conformational change in the bound FN molecules, which results in their linearization and exposure of binding sites for deposition of newly recruited FN molecules and maturation of the fibrils (23, 24, 25). Since FN is one of the earliest matrices assembled and serves as a scaffold for the assembly of other ECM molecules (26, 27, 28, 29), regulation of its fibrillogenesis is crucial for ECM maturation. Some of the ADAMTS family members are known to participate in FN fibrillogenesis, proteolytically cleaving FN (30, 31, 32). Direct cleavage of FN by ADAMTS18 has been suggested by Ataca *et al.* (16), who showed *in vitro* that ADAMTS18 cleaves a recombinant N-terminal 70 kDa FN, leading to the release of a ∼30 kDa fragment. Despite these observations, conclusive identification of full-length FN as an ADAMTS18 substrate and its cleavage sites is still missing, and as such, ADAMTS18 has remained an orphan protein.

The aim of this study was to investigate the role of ADAMTS18 in ECs, validate its substrate, and examine its FN-related mechanism(s). In this work, we quantitated the expression of ADAMTS18 in ECs and assessed its effects on global cellular gene expression and endothelial-related functions following siRNA knockdown. We confirmed FN as a substrate of ADAMTS18, and importantly, using mass spectrometry, we identified two cleavage sites. Furthermore, in ADAMTS18 overexpression studies, we demonstrated that ADAMTS18 proteolytic processing of FN negatively regulates FN fibrillogenesis *in vitro*, which in turn affects cell cycle, adhesion, and proliferation of primary ECs.

## Results

### ADAMTS18 in ECs and the effects of its knockdown

**Figure 1:**
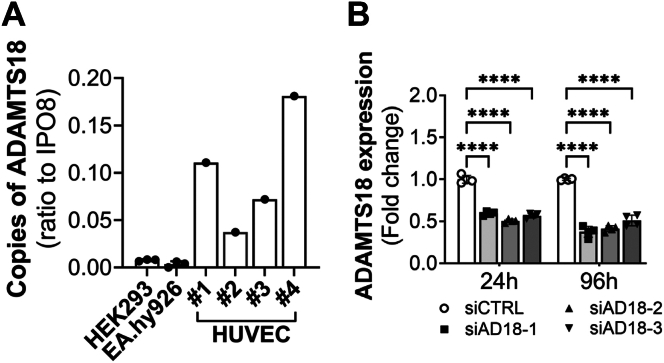
***ADAMTS18* is expressed in HUVECs.***A*, ddPCR analysis of *ADAMTS18* expression of cell lines HEK293 and EA.hy926 and different HUVEC donors at passage 2–3. Replicates for HEK293 and EA.hy926 represent consecutive passages. Expression is represented as a ratio to the reference gene *IPO8*. *B*, analysis of *ADAMTS18* mRNA expression (RT–quantitative PCR) 24 h and 96 h after transfection of HUVECs with siCTRL, siAD18-1, siAD18-2, and siAD18-3 (mean ± SD n = 4, two-way ANOVA, ∗∗∗∗*p* < 0.0001). ADAMTS18, A disintegrin and metalloproteinase with thrombospondin motifs 18; ddPCR, droplet digital PCR; HEK293, human embryonic kidney 293 cell line; HUVEC, human umbilical vein endothelial cell.

**Figure 2:**
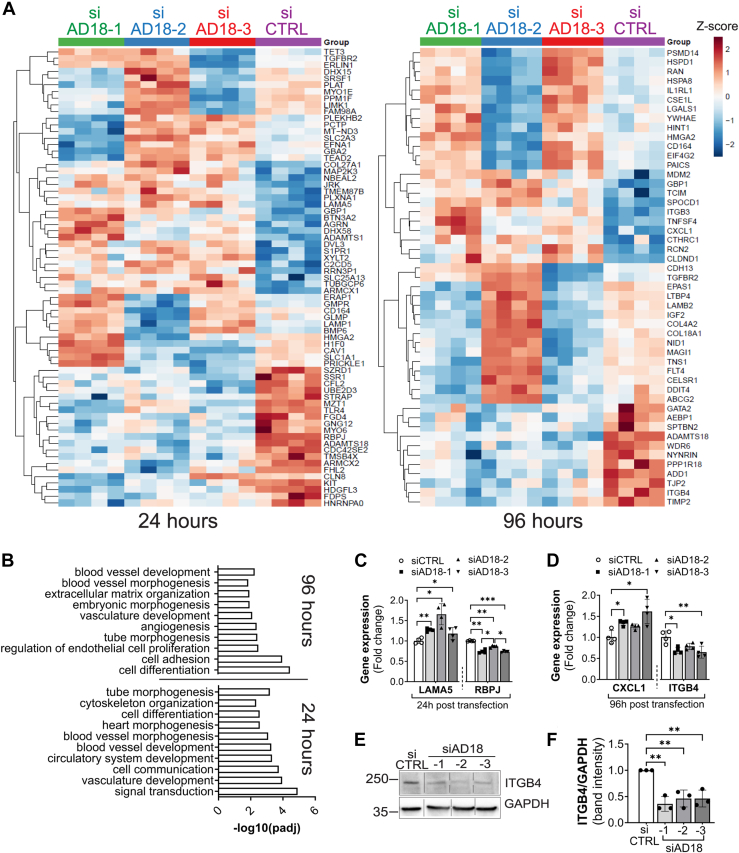
**Effect of *ADAMTS18* knockdown on gene expression.***A*, heatmap representing significant DEGs (*p*_adj_ <0.05) common to siAD18-1, siAD18-2, and siAD18-3 at 24 h (*left*) and 96 h (*right*) after transfection. *B*, Gene Ontology (GO) analysis (g:Profiler) of significant DEGs common to siAD18-1, siAD18-2, and siAD18-3 either at 24 h or 96 h after transfection. *C*, RT–quantitative PCR analysis of *LAMA5* and *RBPJ* expression 24 h after siCTRL, siAD18-1, siAD18-2, and siAD18-3 transfection in HUVEC (mean ± SD, n = 4, lognormal repeated-measures one-way ANOVA with Tukey’s multiple comparison test). *D*, RT–quantitative PCR analysis of *CXCL1* and *ITGB4* expression 96 h after siCTRL, siAD18-1, siAD18-2, and siAD18-3 transfection in HUVEC (mean ± SD, n = 4, lognormal repeated-measures one-way ANOVA with Tukey’s multiple comparison test). *E*, Western blot (WB) analysis of siCTRL-, siAD18-1-, siAD18-2-, and siAD18-3-transfected HUVEC with anti-ITGB4 antibody (1:250 dilution, sc-514426; Santa Cruz) 96 h after transfection. The siCTRL lane was reordered to the left side of the blot from the right side. *F*, densitometry analysis (Image Lab) of ITGB4 205 kDa and GAPDH 36 kDa band intensities. The adjusted volume (Int) for ITGB4 was normalized to an adjusted volume (Int) of GAPDH. The data are presented as fold change of siAD18 to siCTRL (mean ± SD, n = 3 using two different HUVEC donors, ordinary one-way ANOVA). Significance is indicated as follows: ∗*p* < 0.05, ∗∗*p* < 0.01, and ∗∗∗*p* < 0.001. ADAMTS18, A disintegrin and metalloproteinase with thrombospondin motifs 18; DEG, differentially expressed gene; HUVEC, human umbilical vein endothelial cell.

ADAMTS18 was suggested to play a role in endothelial sprouting (13), but its overall effects on ECs are still unknown. To investigate the potential role of ADAMTS18 in ECs, we first assessed its expression profile in primary ECs with droplet digital PCR (ddPCR). We tested human umbilical vein endothelial cells (HUVECs) from different donors and compared ADAMTS18 expression to human embryonic kidney 293 and EA.hy926 cell lines. ADAMTS18 was expressed in all four HUVEC cultures with very little or no expression in the cell lines tested (Fig. 1*A*). The expression of ADAMTS18 in ECs was overall modest and variable between donors. To investigate the role of ADAMTS18 in ECs, we used siRNA knockdown to study its effects on global gene expression and EC-related functions. The three tested siRNAs targeting *ADAMTS18* (siAD18-1, siAD18-2, and siAD18-3) significantly reduced 50% of *ADAMTS18* mRNA at 24 h and 96 h after transfection as compared with siRNA-control–treated cells (Fig. 1*B*). Next, we evaluated the effect of *ADAMTS18* knockdown on global mRNA expression by bulk RNA-Seq. To overcome donor variability, pooled HUVECs from three donors were used for each transfection in these studies. Significant (*p* adjusted value [*p*_adj_] <0.05) differentially expressed genes (DEGs) that were identified in all siRNA groups targeting *ADAMTS18* are represented in the heatmap (Fig 2*A*). Importantly, one of these DEGs was *ADAMTS18*, which confirmed the siRNA knockdown in these samples (Fig. 2*A*). More DEGs were observed at 24 h (69 DEGs, Table S1) *versus* 96 h (49 DEGs, Table S2) following *ADAMTS18* siRNA knockdown, with only four genes, in addition to *ADAMTS18*, common to both time points (*CD164*, *HMGA2*, *TGFBR2*, and *GBP1*). Despite this, the DEGs were predicated to impact similar processes in the Gene Ontology (GO) analysis. Common processes were related to blood vessel development and morphogenesis, vasculature development, tube morphogenesis, and cell differentiation (Fig. 2*B*). Interestingly, at 96 h, GO analysis identified cell adhesion and ECM organization as affected processes (Fig. 2*B*). These were the same GO processes that were identified when the analysis was performed with only siAD18-1 and siAD18-3 as siAD18-2 was more different, suggesting off-target effects (Fig. S1).

Overall, *ADAMTS18* knockdown had a moderate effect on gene expression in ECs such that only a few significant genes had a log2 fold change (FC) >0.2 or <-0.2 (Tables 1 and 2, for 24 and 96 h, respectively). Some of these genes were confirmed by RT–quantitative PCR (qPCR), including *LAMA5* (laminin α5) and *RBPJ* from 24 h samples (Fig. 2*C*) as well as *CXCL1* and *ITGB4* (integrin β4) from 96 h samples (Fig. 2*D*). *ITGB4* was of particular interest, as this gene is important for endothelial adhesion. Further analysis of siRNA-transfected cells demonstrated reduction of ITGB4 at 96 h at the protein level as determined by Western blot (WB; Fig. 2*E*), consistent with the observed *ITGB4* mRNA downregulation. Densitometry analysis (Image Lab) of ITGB4 bands demonstrated significant reduction in ITGB4 protein (Fig. 2*F*).

**Table 1:** Significant DEGs (*p*_adj_ <0.05 and log2FC <−0.2 or >0.2) identified in all the three siRNAs targeting ADAMTS18 24 h after transfection of HUVECs

Gene name	siAD18-1		siAD18-2		siAD18-3	
	log2FC	*p*_adj_	log2FC	*p*_adj_	log2FC	*p*_adj_
*ADAMTS18*	−0.57	1.39E-07	−0.96	6.81E-16	−0.83	3.16E-11
*RBPJ*	−0.41	1.83E-09	−0.54	1.44E-16	−0.38	2.00E-08
*KIT*	−0.20	2.08E-03	−0.30	8.01E-06	−0.51	7.82E-13
*DHX58*	0.67	8.49E-07	0.24	1.25E-02	1.70	3.24E-19
*ARMCX1*	0.25	4.70E-04	0.25	2.52E-04	0.21	2.14E-03
*NBEAL2*	0.24	8.80E-05	0.25	2.81E-05	0.22	2.88E-04
*TGFBR2*	−0.45	6.15E-34	0.26	4.82E-12	0.25	2.91E-11
*LAMA5*	0.34	2.63E-04	0.31	2.08E-04	0.20	9.26E-03

**Table 2:** Table of significant DEGs (*p*_adj_ <0.05 and log2FC <−0.2 or >0.2) identified in all the three siRNAs targeting ADAMTS18 96 h after transfection of HUVECs

Gene name	siAD18-1		siAD18-2		siAD18-3	
	log2FC	*p*_adj_	log2FC	*p*_adj_	log2FC	*p*_adj_
*ADAMTS18*	−1.24	2.18E-22	−0.55	2.34E-07	−0.76	1.00E-09
*IGF2*	−0.35	1.28E-11	0.26	1.18E-06	−0.33	5.36E-10
*ITGB4*	−0.31	1.56E-05	−0.44	1.43E-07	−0.45	2.27E-07
*FLT4*	−0.22	1.78E-05	0.28	3.81E-07	−0.34	5.43E-10
*TCIM*	0.21	1.21E-03	0.44	9.07E-08	0.24	1.67E-03
*IL1RL1*	0.31	1.12E-09	−0.21	1.25E-04	0.33	1.48E-09
*CXCL1*	0.51	2.24E-06	0.20	2.06E-02	0.79	5.24E-09

**Figure 3:**
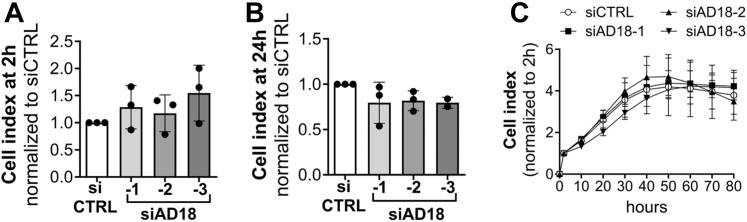
***ADAMTS18* knockdown did not affect adhesion, migration, or proliferation.***A*, cell adhesion assay using the XCelligence DP system for siCTRL, siAD18-1, siAD18-2, and siAD18-3 transfected HUVEC plated 96 h after transfection. Adhesion was assessed 2 h after plating, and data were normalized to siCTRL (mean ± SD, n = 3, Kruskal–Wallis test, not significant). *B*, cell migration assay using the XCelligence DP system for siCTRL, siAD18-1, siAD18-2, and siAD18-3 transfected HUVEC plated 96 h after transfection. Migration was assessed 24 h after plating, and data were normalized to siCTRL (mean ± SD, n = 3, Kruskal–Wallis test, not significant). *C*, cell proliferation assay using the XCelligence DP system for siCTRL, siAD18-1, siAD18-2, and siAD18-3 transfected HUVEC. The data show cell index normalized to 2 h over 80 h (mean ± SD, n = 3, two-way ANOVA, not significant). ADAMTS18, A disintegrin and metalloproteinase with thrombospondin motifs 18; HUVEC, human umbilical vein endothelial cell.

Since we observed changes in expression of adhesion molecules (ITGB4, LAMA5) following ADAMTS18 knockdown, we then investigated the adhesion and migration of si-AD18-treated HUVECs. Although there was a trend for increased adhesion (Fig. 3*A*) and decreased migration (Fig. 3*B*) of si-AD18-transfected HUVEC, these changes were not significant, perhaps because of insufficient knockdown of *ADAMTS18* in all the ECs. Likewise, no effect on HUVEC proliferation was measured following *ADAMTS18* knockdown (Fig. 3*C*).

#### Overexpression of ADAMTS18 and ADAMTS18-E437A

**Figure 4:**
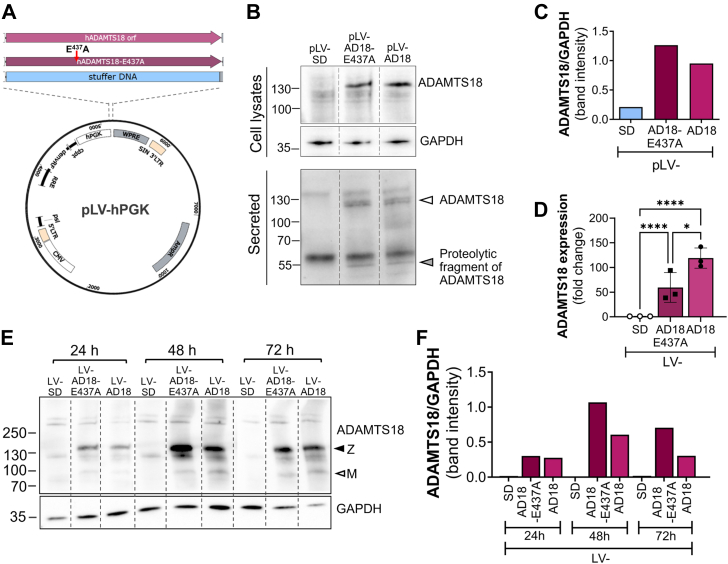
**Vector generation and testing.***A*, pLV-hPGK, plasmid map with indicated inserts used to generate vectors pLV-AD18, pLV-AD18-E437A, and pLV-SD. *B*, Western blot analysis of cell lysates and conditioned media from 293T cells transfected with pLV-SD, pLV-AD18-E437A, and pLV-AD18, 72 h after transfection, with anti-ADAMTS18 antibody (from Dr Cathrin Brisken's lab). *Empty arrow* indicates ADAMTS18 and mutant ADAMTS18, whereas *gray arrow* indicates a proteolytic fragment of ADAMTS18. *C*, densitometry analysis of ADAMTS18 band intensity from 293T lysates. The adjusted volume (Int) for ADAMTS18 was normalized to an adjusted volume (Int) of GAPDH. *D*, RT–quantitative PCR analysis of ADAMTS18 or ADAMTS18-E437A expression in LV-SD–, LV-AD18-E437A–, and LV-AD18–transduced HUVEC at 72 h (mean ± SD, n = 3, lognormal ordinary one-way ANOVA, ∗*p* < 0.05, ∗∗∗∗*p* < 0.0001). *E*, Western blot analysis of cell lysates from LV-SD–, LV-AD18-E437A–, and LV-AD18–transduced HUVEC at 24 h, 48 h, and 72 h with anti-ADAMTS18 antibody (from Dr Cathrin Brisken's lab). The *arrows* represent ADAMTS18 zymogen (Z, *black arrow*) and mature forms arising from expected Furin processing (M, *empty arrow*). *F*, densitometry analysis of ADAMTS18 band intensity from HUVEC lysates. The adjusted volume (Int) for ADAMTS18 was normalized to an adjusted volume (Int) of GAPDH. ADAMTS18, A disintegrin and metalloproteinase with thrombospondin motifs 18; HUVEC, human umbilical vein endothelial cell.

To further investigate the potential role of ADAMTS18 in primary ECs and its molecular mechanism, we aimed to overexpress it in HUVECs. As HUVECs are poorly transfected by plasmid transfection, we utilized lentiviral vectors (LVs), which efficiently transduce cells *in vitro* (13), to overexpress ADAMTS18. In addition to the wildtype ADAMTS18, we generated a mutant ADAMTS18, based on previous reports of inactivating mutations of the catalytic site of ADAMTS16 (30) and ADAMTS9 (31), by single-point mutation of the corresponding catalytic site in ADAMTS18 at E437A. We cloned and produced LVs encoding either ADAMTS18 (LV-AD18) or ADAMTS18-E437A (LV-AD18-E437A) or then a stuffer DNA (SD) to control for vector length (Fig. 4*A*). Developed vector plasmids were verified for ADAMTS18 expression and protein production following transfection of 293T. ADAMTS18 protein was detected in the transfected cell lysates and secreted into the conditioned media (Fig. 4*B*). Densitometry analysis of ADAMTS18 bands from cell lysates clearly showed the increase in wildtype and mutant ADAMTS18 compared with control (Fig. 4*C*). The secreted ADAMTS18 was observed both as the mature (*empty arrow*) and a proteolytic fragment (*gray arrow*) of ADAMTS18 (Fig 4*B*, *lower panel*). To test the newly generated LVs, HUVECs were transduced with LV-AD18, LV-AD18-E437A, and LV-SD. RT–qPCR analysis showed a significant increase in wildtype and mutant ADAMTS18 mRNA expression after 72 h in LV-AD18– and LV-AD18-E437A–transduced cells, respectively (Fig. 4*D*). WB analysis of cellular lysates after 24 h, 48 h, and 72 h confirmed overexpression of both ADAMTS18 and ADAMTS18-E437A at the protein level. Clear bands at approximately 135 kDa and 100 kDa were observed at all time points; these are likely to be the zymogen (Z, *black arrow*) and mature (M, *empty arrow*) forms of ADAMTS18, respectively (Fig. 4*E*), which arise from Furin processing (8). The maximal ADAMTS18 protein was observed at 48 h following transduction (Fig. 4*E*). Interestingly, increased amounts of ADAMTS18 were detected in cells expressing the mutant ADAMTS18 (Fig. 4*F*), despite significantly lower mRNA expression (Fig. 4*D*). This is unlikely to be due to altered secretion since both ADAMTS18 and mutant ADAMTS18 are secreted by transfected cells (Fig. 4*B*).

#### Overexpression of ADAMTS18 affects EC cycle and proliferation

**Figure 5:**
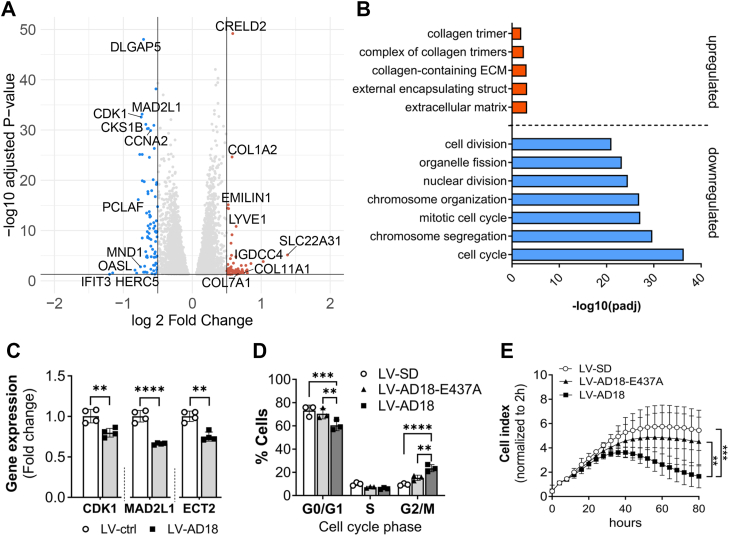
**Overexpression of ADAMTS18 affects endothelial cell cycle and proliferation.***A*, Volcano plot representing DEGs (*p*_adj_ <0.05, 0.5 <log2fold change [FC] <−0.5) for LV-AD18–transduced cells compared with LV-ctrl (LV-GFP). Some of the DEGs of interest are highlighted. *B*, Gene Ontology (GO) analysis (g:Profiler) of upregulated and downregulated genes in LV-AD18 cells. *C*, RT–quantitative PCR analysis of *CKD1*, *MAD2L1*, and *ECT2* expression from LV-ctrl (LV-GFP) and LV-AD18 cultures 72 h after transduction (mean ± SD, n = 4, lognormal *t* test). *D*, cell cycle analysis of HUVEC transduced with LV-SD, LV-AD18-E437A, and LV-AD18. The analysis was performed with NucleoCounter NC-3000 using the Two-Step Cell Cycle Assay (mean ± SD, n = 3, two-way ANOVA, differences between LV-SD and LV-AD18-E437A are not significant). *E*, cell proliferation assay using the XCelligence DP system for LV-SD–, LV-AD18-E437A–, and LV-AD18–transduced HUVEC. The data show cell index normalized to 2 h over 80 h (mean ± SD, n = 3, two-way ANOVA, all time points after and including 48 h for LV-SD *versus* LV-AD18 and 60 h for LV-AD18-E437A *versus* LV-AD18 were significantly different; no significant differences between LV-SD and LV-AD18-E437A). Statistical significance is indicated as follows: ∗∗*p* < 0.01, ∗∗∗*p* < 0.001, and ∗∗∗∗*p* < 0.0001. ADAMTS18, A disintegrin and metalloproteinase with thrombospondin motifs 18; DEG, differentially expressed gene; HUVEC, human umbilical vein endothelial cell.

To study the impact of overexpression of ADAMTS18 on ECs, we first assessed its effects on global mRNA expression by bulk RNA-Seq at 72 h after transduction. ADAMTS18 overexpression had a moderate effect on gene expression in ECs, as only 74 and 86 genes were significantly upregulated (*p*_adj_ <0.05, log2FC >0.5, Table 3) or downregulated (*p*_adj_ <0.05, log2FC <−0.5, Table 4), respectively (Fig. 5*A*). GO analysis revealed that most of the upregulated genes following ADAMTS18 overexpression were ECM associated and collagen matrix–related genes, whereas the downregulated genes were related to cell division and cell cycle (Fig. 5*B*). We confirmed by RT–qPCR significant downregulation of *CDK1*, *MAD2L1*, and *ECT2*, genes that are related to cell cycle (Fig. 5*C*).

**Table 3:** Table of significant DEGs (*p*_adj_ <0.05) upregulated (log2FC >0.5) following overexpression of ADAMTS18 in HUVECs

Gene name	log2FC	*p*_adj_	Gene name	log2FC	*p*_adj_
*SLC22A31*	1.38	7.18E-06	PRSS53	0.58	7.00E-03
*IGDCC4*	1.03	1.46E-04	SCNN1D	0.58	1.48E-02
*AC006128.1*	0.85	3.61E-04	UGDH-AS1	0.58	4.42E-02
*MAST1*	0.80	2.87E-02	SLC25A25-AS1	0.58	4.17E-02
*AP000892.3*	0.80	6.50E-03	COL1A2	0.58	2.32E-25
*INHBB*	0.79	1.10E-02	ATG16L2	0.58	6.89E-10
*OLFML2A*	0.78	1.08E-02	PNMA6A	0.57	8.84E-03
*PARM1*	0.78	8.60E-04	AHRR	0.57	8.27E-06
*COL11A1*	0.74	2.71E-02	PPARGC1B	0.56	1.62E-03
*PDPK2P*	0.73	1.29E-02	MEG3	0.56	3.27E-08
*CHPF*	0.73	1.88E-02	ALDH3B1	0.56	4.69E-02
*MST1P2*	0.72	5.63E-03	LIME1	0.56	3.54E-03
*CARNS1*	0.72	1.39E-02	AC069281.2	0.55	3.89E-05
*GPNMB*	0.71	8.92E-04	CATSPER2P1	0.55	1.10E-02
*ZNF710-AS1*	0.71	2.57E-02	HSP90B1	0.55	1.34E-111
*AL096870.2*	0.71	2.02E-02	FER1L4	0.55	2.63E-03
*AL591845.1*	0.69	3.45E-02	BBS1	0.55	4.40E-03
*QPRT*	0.68	1.88E-02	AC084018.1	0.55	4.96E-02
*AC139887.2*	0.68	1.04E-02	RTEL1-TNFRSF6B	0.55	5.13E-03
*COL7A1*	0.67	2.65E-02	AC006001.3	0.54	4.84E-03
*AL121753.2*	0.67	3.79E-02	AC234582.1	0.54	3.51E-02
*MT-TF*	0.67	1.94E-02	AC124798.1	0.54	3.15E-03
*ASMTL-AS1*	0.66	3.99E-03	GARNL3	0.53	2.85E-02
*SELPLG*	0.65	1.22E-02	LRP1	0.53	4.30E-15
*SSC5D*	0.65	2.69E-02	LPAR2	0.53	1.41E-02
*ODF3B*	0.65	4.79E-02	PDIA4	0.53	1.16E-101
*LYVE1*	0.64	1.48E-11	MROH6	0.52	2.72E-02
*STC1*	0.63	5.93E-03	EMILIN1	0.52	7.43E-16
*ASPHD1*	0.63	1.61E-05	ATG9B	0.52	4.78E-02
*DIO2*	0.63	3.34E-02	MT-TE	0.52	5.43E-04
*LINC00854*	0.62	2.49E-02	EGFL8	0.52	4.30E-02
*SOX8*	0.62	1.16E-02	CPM	0.52	1.17E-02
*ERICD*	0.61	2.13E-02	SYT16	0.51	4.02E-02
*PTOV1-AS2*	0.60	3.02E-04	SH2D5	0.51	4.17E-03
*CNN1*	0.59	1.00E-02	MAP1A	0.51	3.54E-15
*CRELD2*	0.59	6.40E-50	NOV	0.51	1.11E-02
*CXCL12*	0.58	1.27E-02	AC000403.1	0.50	1.06E-02

**Table 4:** Table of significant DEGs (*p*_adj_ <0.05) downregulated (log2FC <−0.5) following overexpression of ADAMTS18 in HUVECs

Gene name	log2FC	*p*_adj_	Gene name	log2FC	*p*_adj_
*IFIT3*	−1.20	4.81E-02	*RTKN2*	−0.60	4.95E-03
*OASL*	−1.15	2.69E-02	*KIF15*	−0.60	1.24E-07
*TMSB15A*	−0.83	7.36E-03	*ESCO2*	−0.60	5.93E-09
*HERC5*	−0.81	2.97E-02	*POLE2*	−0.60	1.59E-05
*PCLAF*	−0.78	6.98E-17	*CCNB2*	−0.59	1.14E-18
*TNFSF18*	−0.76	7.24E-26	*TLR3*	−0.59	3.78E-02
*MND1*	−0.75	1.63E-03	*CCNE2*	−0.58	5.44E-07
*CDK1*	−0.74	2.67E-33	*BUB1B*	−0.57	7.61E-21
*MAD2L1*	−0.73	7.04E-34	*DSCC1*	−0.56	7.75E-04
*DEPDC1B*	−0.73	3.06E-09	*WDR76*	−0.56	3.62E-11
*DEPDC1*	−0.73	7.24E-26	*SNRPGP2*	−0.56	9.77E-03
*HMMR*	−0.73	1.24E-20	*CENPW*	−0.56	2.67E-12
*DLGAP5*	−0.71	9.34E-49	*CCNB1*	−0.56	1.17E-31
*PLSCR1*	−0.71	1.88E-02	*SGO1*	−0.55	2.09E-05
*PBK*	−0.70	1.87E-20	*BIRC5*	−0.55	4.65E-27
*MTCP1*	−0.70	1.90E-02	*CDC25C*	−0.55	8.02E-04
*FANCB*	−0.69	1.23E-03	*NEK2*	−0.54	4.02E-06
*CXCL11*	−0.68	1.78E-02	*SLC2A12*	−0.54	4.69E-02
*PRR15*	−0.68	1.54E-06	*CENPU*	−0.54	1.25E-11
*NCAPG*	−0.67	7.48E-32	*CHAC2*	−0.54	1.74E-06
*APOBEC3B*	−0.66	1.78E-05	*CDC6*	−0.54	1.80E-17
*TTK*	−0.66	3.31E-14	*SKA3*	−0.54	1.53E-10
*MYBL1*	−0.66	1.09E-05	*PIMREG*	−0.53	1.49E-08
*ACKR4*	−0.66	2.14E-02	*NCAPH*	−0.53	1.80E-10
*CKS1B*	−0.65	5.48E-31	*CENPQ*	−0.53	2.90E-04
*OIP5*	−0.65	2.23E-05	*BARD1*	−0.53	1.84E-05
*SPC25*	−0.65	1.62E-10	*SGO2*	−0.53	1.33E-10
*CDKN3*	−0.65	2.56E-09	*TOP2A*	−0.53	6.62E-39
*RAD51AP1*	−0.65	1.52E-09	*UBE2T*	−0.52	2.30E-10
*GINS1*	−0.64	1.75E-18	*AC099850.3*	−0.52	3.74E-04
*GMNN*	−0.64	4.80E-18	*CDC45*	−0.52	1.13E-12
*PARPBP*	−0.64	1.13E-11	*CDC20*	−0.52	6.71E-20
*NUSAP1*	−0.64	4.81E-31	*EME1*	−0.52	6.06E-03
*EXO1*	−0.63	3.57E-10	*CKS2*	−0.52	2.84E-20
*CENPA*	−0.62	3.63E-09	*AC008560.1*	−0.51	2.29E-02
*CENPK*	−0.62	1.72E-14	*MYCBP*	−0.51	1.67E-10
*BUB1*	−0.62	2.75E-25	*CLGN*	−0.51	9.31E-03
*NUF2*	−0.62	3.64E-11	*GINS2*	−0.51	1.80E-09
*NDC80*	−0.62	7.87E-14	RGS2	−0.51	1.20E-05
*CKAP2L*	−0.62	2.05E-13	SPATA24	−0.51	2.14E-03
*FANCD2*	−0.61	1.48E-13	RPL39P3	−0.51	1.96E-02
*FAM111B*	−0.61	5.18E-12	KIF20A	−0.50	1.74E-15
*CCNA2*	−0.60	1.24E-30	ANLN	−0.50	4.80E-40

Since many of the downregulated genes following overexpression of ADAMTS18 related to cell division, we performed cell-cycle analysis of LV-transduced cells 72 h after transduction. HUVECs overexpressing ADAMTS18 demonstrated a significant increase in the percentage of cells in G2/M phase and a significant decrease in G1/G0 phase (Fig. 5*D*), indicating that ADAMTS18 overexpression leads to cell cycle arrest in G2/M, preventing cells from completing their division to generate daughter cells. The effect of ADAMTS18 on cell cycle was also evident in the proliferation of HUVECs transduced with LV-AD18, which demonstrated significantly decreased cell proliferation compared with LV-AD18-E437A– and LV-SD–transduced cells starting from 40 h (Fig. 5*E*). This is consistent with the observed block in cell cycle and taken together demonstrate an effect of ADAMTS18 on cell division.

#### ADAMTS18 impairs FN fibrillogenesis

**Figure 6:**
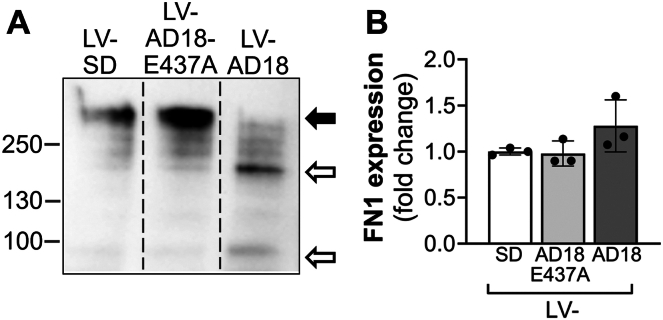
**ADAMTS18 proteolytically cleaves FN.***A*, Western blot analysis of conditioned media from LV-SD–, LV-AD18-E437A–, and LV-AD18–transduced HUVEC at 24 h with anti-FN antibody (#PA5-29578; Invitrogen). The *arrows* represent FN full length (*black arrow*) and FN proteolytic fragments (*empty arrow*). *B*, quantitative RT–PCR analysis of *FN1* expression in LV-SD–, LV-AD18-E437A–, and LV-AD18–transduced HUVEC 72 h after transduction (mean ± SD, n = 3, lognormal ordinary one-way ANOVA, not significant). ADAMTS18, A disintegrin and metalloproteinase with thrombospondin motifs 18; FN, fibronectin; HUVEC, human umbilical vein endothelial cell.

HUVECs are routinely cultured on FN–gelatin coating, and ADAMTS18 has been implicated in FN cleavage (10, 14, 15, 16). Since cell proliferation can be affected by cell adhesion, we reasoned that a detrimental effect of ADAMTS18 on FN coating could lead to cellular detachment and thus affect cellular proliferation. To test the effect of ADAMTS18 on FN, LV-AD18–, LV-AD18-E437A–, or LV-SD–transduced HUVECs were seeded on FN-coated plates, and conditioned media were analyzed by WB after 24 h (Fig. 6*A*). The full-length FN (270 kDa, *black arrow*) was detected in high abundance in the media from LV-AD18-E437A– and LV-SD–transduced cells, in contrast to LV-AD18–transduced cells, which showed a reduced amount of 270 kDa FN. Instead, smaller FN fragments of approximately 200 kDa and 95 kDa (*empty arrows*) were detected at higher levels in LV-AD18 media compared with the controls, supporting that ADAMTS18 is involved in proteolytic processing of FN. We observed a lower amount of full-length FN in the conditioned medium of ADAMTS18-transduced cells with a corresponding increase in proteolytically cleaved FN fragments, which is not because of regulation of FN expression by the cells; as on the contrary, this group appeared to express slightly higher, albeit not significant, *FN1* mRNA (Fig. 6*B*).

**Figure 7:**
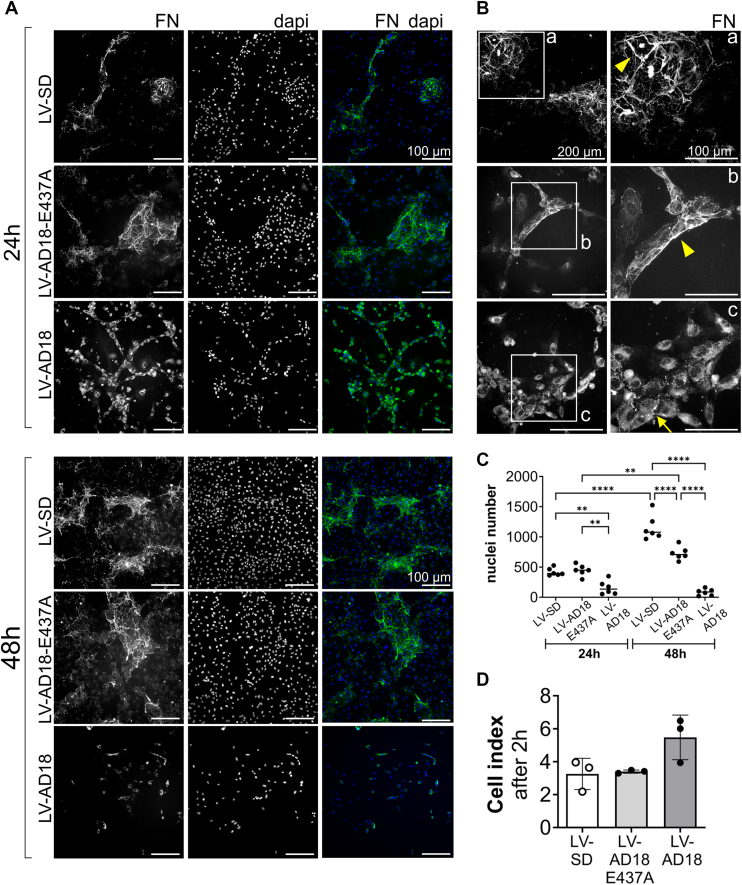
**ADAMTS18 regulates FN fibrillogenesis**. Immunofluorescent images of deposited FN matrix at 24 h and 48 h in LV-SD–, LV-AD18-E437A–, and LV-AD18–transduced HUVEC cultures. Fixed cells were immunostained with anti-FN antibody (#PA5-29578). Pictures were taken with a Nikon Eclipse NIE and DS-Ri2 camera at 10× magnification for 24 h and 48 h cultures (*A*) or 20× magnification for 24 h cultures (*B*). On the *far right*, enlargements of FN matrix pictures are indicated by the *white box inserts*. The *yellow arrowheads* indicate FN fibrils, and *yellow arrows* indicate punctate FN staining. *C*, numeration of cell nuclei (using ImageJ) at 24 h and 48 h (mean ± SD, n = 6 images per group, two-way ANOVA, ∗∗*p* < 0.01 and ∗∗∗∗*p* < 0.0001). *D*, cell adhesion assay using the XCelligence DP system for LV-SD–, LV-AD18-E437A–, and LV-AD18–transduced HUVECs plated 72 h after transduction. Adhesion was assessed 2 h after plating (mean ± SD, n = 3, ordinary one-way ANOVA, not significant). ADAMTS18, A disintegrin and metalloproteinase with thrombospondin motifs 18; Dapi, 4′,6-diamidino-2-phenylindole; FN, fibronectin; HUVEC, human umbilical vein endothelial cell.

To determine the consequence of ADAMTS18 processing of FN on the ECM, LV-SD, LV-AD18-E437A, and LV-AD18 cultures seeded on FN-coated plates were immunostained for FN. Reduction in FN matrix deposition was observed at 24 h in ADAMTS18-overexpressing cultures, contrary to LV-AD18-E437A– and LV-SD–transduced cells (Fig. 7*A*). In these latter cultures, FN fibrils also differed in their appearance, as FN was characterized by strand-like structures that were visible by 24 h and increased following 48 h (Fig. 7*A*). In contrast, FN in ADAMTS18 cultures appeared diffuse and punctate as seen at higher magnification (Fig. 7*B*) and appeared to localize in the cytoplasm. Furthermore, despite equal cell plating, we observed a significant reduction in cell numbers in LV-AD18 cultures compared with LV-AD18-E437A and LV-SD, as determined by counting of 4′,6-diamidino-2-phenylindole–stained nuclei at both time points (Fig. 7*C*). This correlates the ADAMTS18 effects on FN with reduced cell numbers and is in line with the previously observed reduction in proliferation presented in Figure 5. This decrease in cell nuclei became even more drastic at 48 h for LV-AD18 culture; in contrast, an increase in cell number was observed for LV-AD18-E437A and LV-SD cultures (Fig. 7*C*). Interestingly, at this later time point, fewer nuclei were also noted in LV-AD18-E437A cultures compared with LV-SD, which may indicate residual activity of this mutant. Of course, the decreased cell number in ADAMTS18-overexpressing cells may be due to reduced adhesion; however, this was not the case, as ADAMTS18-overexpressing cells demonstrated moderately higher, albeit not significant, adhesion compared with the mutant or control transduced cells (Fig. 7*D*).

**Figure 8:**
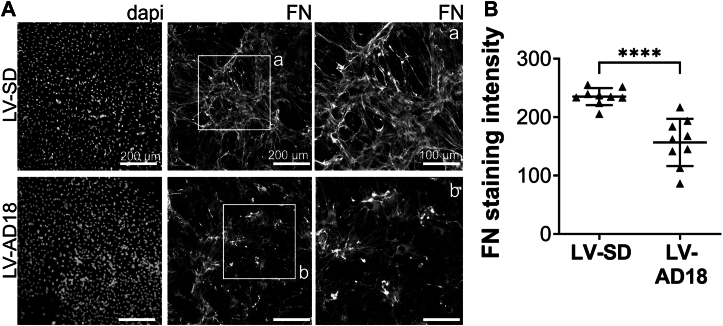
**ADAMTS18 impairs FN fibrillogenesis in HUVEC and fibroblast cocultures**. *A*, immunofluorescent images of deposited FN matrix at day 6 of LV-SD– and LV-AD18–transduced HUVECs cocultured with HPFc. Fixed cells were immunostained with anti-FN antibody (#PA5-29578). Pictures were taken with the Nikon Eclipse NIE and DS-Ri2 camera at 10× magnification. On the *right*, enlargements of the FN matrix that are indicated by the *white box inserts* a and b. *B*, quantification of FN staining in LV-SD– and LV-AD18–transduced HUVECs cocultured with HPFc. Quantification was performed with ImageJ (brightness adjusted to 45–248, threshold adjusted at 0–220), and mean intensity was graphed (mean ± SD, n = 9 images unpaired *t* test, ∗∗∗∗*p* < 0.0001). ADAMTS18, A disintegrin and metalloproteinase with thrombospondin motifs 18; Dapi, 4′,6-diamidino-2-phenylindole; FN, fibronectin; HPFc, human pulmonary fibroblast; HUVEC, human umbilical vein endothelial cell.

Investigation of ADAMTS18 effect on FN using HUVECs was hampered by the loss of cells; therefore, we cocultured LV-transduced HUVECs with primary fibroblasts, which abundantly secrete FN, to further study the effect of ADAMTS18 on FN fibrillogenesis. Our goal was to understand if ADAMTS18 secreted by ECs could affect FN secreted by fibroblasts in a coculture setting to resemble more the complexity of a tissue microenvironment. HUVECs transduced with LV-SD or LV-AD18 were cocultured with primary human pulmonary fibroblasts (HPFcs) for 6 days. FN immunostaining revealed that ADAMTS18-overexpressing cocultures had reduced FN matrix deposited compared with LV-SD cells, as can be seen in the microscope pictures (Fig. 8*A*), and was also confirmed by quantification of FN staining (Fig. 8*B*). In addition, despite abundant FN in the cocultures of LV-AD18 and HPFc, punctate deposition of FN was clearly visible in this group, and fewer FN fibrils were observed, in contrast to the control group, where FN was mainly found in fibrils (Fig. 8*A*).

#### Identification of two cleavage sites of FN by ADAMTS18

**Figure 9:**
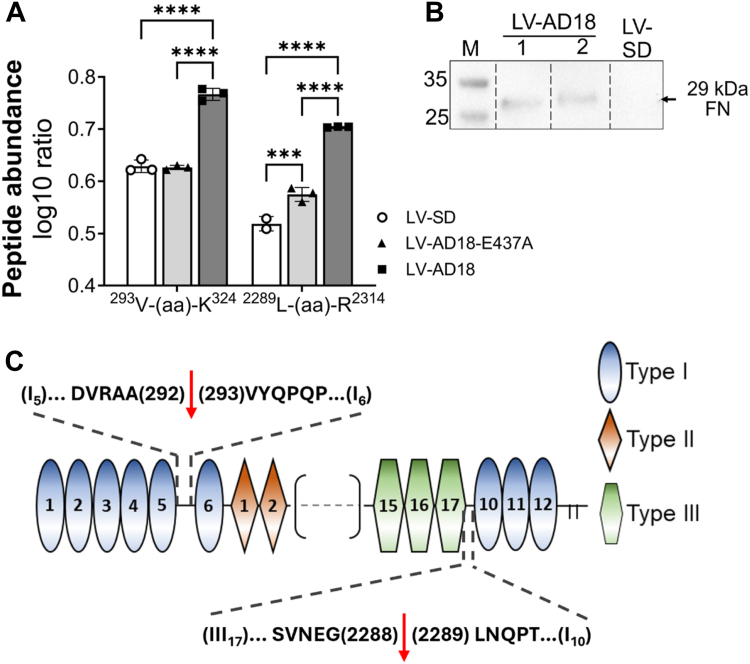
**Identification of FN cleavage sites by ADAMTS18.***A*, histograms representing relative abundance of the peptides 293-VYQPQPHPQPPPYGHCVTDSGVVYSVGMQWLK-324 and 2289-LNQPTDDSCFDPYTVSHYAVGDEWER-2314, according to mass spectrometry identification (normalized to log10 of total FN abundance, mean ± SD, n = 3, two-way ANOVA, ∗∗∗*p* < 0.001, ∗∗∗∗*p* < 0.0001). *B*, Western blot analysis of concentrated conditioned media (serum free) from LV-SD and two different LV-AD18 cultures at 24 h after seeding on FN coating. The *arrow* indicates the N-terminal 29 kDa FN fragment generated by ADAMTS18 proteolytic cleavage of FN in the linker region FN-I_5_-I_6_, detected with an N-terminal anti-FN monoclonal antibody (#ab270240). *C*, cartoon representing FN sequence, with focus on identified ADAMTS18 cleavage sites. The *red arrows* in the expansion of FN linker regions I_5_-I_6_ and III_17_-I_10_ sequences indicate the cleavage sites. ADAMTS18, A disintegrin and metalloproteinase with thrombospondin motifs 18; FN, fibronectin.

Despite the clear evidence of ADAMTS18 proteolytic processing of FN, identification of the cleavage sites was still unresolved. Therefore, we analyzed conditioned media from ADAMTS18-overexpressing cells with mass spectrometry to determine FN cleavage sites. LV-SD–, LV-AD18-E437A–, and LV-AD18–transduced HUVECs were cultured on FN for 24 h in serum-free medium. The total protein from conditioned media was digested with Lys-C and trypsin and analyzed with mass spectrometry. Lys-C and trypsin cleave proteins from the C-terminal side of lysine and arginine residues; therefore, peptides generated by cleavages other than at the C-terminal side of lysine and arginine should be considered generated by some other proteases. Mass spectrometry analysis detected a FN peptide ^293^VYQPQPHPQPPPYGHCVTDSGVVYSVGMQWLK^324^, more highly abundant in ADAMTS18 samples compared with ADAMTS18-E437A and SD samples (Fig. 9*A*). This peptide, located in the N-terminal linker region FN-I_5-6_, is generated by cleavage between Alanine^292^ and Valine^293^, which cannot be catalyzed by Lys-C or trypsin. This cleavage is expected to generate a 29 kDa FN fragment, which was detected in the conditioned media from ADAMTS18 cells but not from control cultures by WB using an FN N-terminal antibody (33) (Fig. 9*B*). The percentage of cleaved peptide compared with the parent peptide ^291^AAVYQPQPHPQPPPYGHCVTDSGVVYSVGMQWLK^324^ was 34% in ADAMTS18 samples as compared with 1% for ADAMTS18-E437A or SD control samples, strongly suggesting that the cleavage is catalyzed by ADAMTS18. Furthermore, the peptide ^2289^LNQPTDDSCFDPYTVSHYAVGDEWER^2314^, located in the C-terminal linker region FN-III_17_-I_10_, was generated by cleavage between Glycine^2288^ and Leucine^2289^ and was also detected at higher amounts in ADAMTS18 conditioned media (Fig. 9*A*). As this cleavage cannot be generated by Lys-C or trypsin, then this confirms catalysis by a different protease. Interestingly, this second fragment was also found in significantly higher amounts in ADAMTS18-E437A conditioned media compared with SD, suggesting some residual catalytic activity in the mutant (Fig. 9*A*). The presence of these two peptides in high abundance in ADAMTS18 samples but not in ADAMTS18-E437A or SD controls suggests that they are generated by the ADAMTS18 cleavages of FN between A^292^–V^293^ and G^2288^–L^2289^ (Fig. 9*C*).

## Discussion

In our previous studies, we had reported that *ADAMTS18* is regulated by the endothelial-specific superenhancer SE12313 and that siRNA knockdown of *ADAMTS18* had a detrimental effect on endothelial sprouting *in vitro* (13). These findings indicated an uncharacterized function of ADAMTS18 in ECs, but the mechanism remained unknown. In this study, we show that ADAMTS18 deficiency in HUVECs alters expression of genes relating to vessel development and cell adhesion, such as ITGB4. Whereas overexpression of ADAMTS18 delineated a role for this enzyme in the proteolytic processing of the FN molecule and the regulation of FN fibrillogenesis. These activities impacted EC proliferation and are likely to affect sprouting, as remodeling of the ECM is important for angiogenesis (34).

The results of our study collectively support that ADAMTS18 negatively regulates FN assembly by cleaving FN and preventing fibril formation, thereby contributing to the remodeling of the ECM. In WB analysis, we demonstrated cleavage of FN by ADAMTS18, and importantly, using mass spectrometry, we identified that ADAMTS18 cleaves FN specifically at A^292^–V^293^ in between the FN-I_5_ and FN-I_6_ linker region to generate the N-terminal region FN-I_1–5_ (29 kDa), which is known to be essential for FN fibrillogenesis (23, 24, 25). Schwarzbauer *et al*. (25) showed that recombinant FN molecules lacking the N-terminal I_1–5_ did not assemble into FN matrix, as they did not bind other FN molecules. These results lead the authors to conclude that FN-I_1–5_ must contain an FN–FN binding site essential for FN fibrillogenesis. Other studies demonstrated that this fragment can also bind to cells in monolayer and thereby inhibit FN fibrillogenesis (23, 35). Our immunostaining analysis clearly demonstrated that in ADAMTS18-overexpressing cells, FN fibrillogenesis was almost completely blocked, and the FN appeared punctate. FN fibril formation begins with dimeric FN binding to cell membrane integrins through the RGD site; this is followed by FN–FN intermolecular interactions to assemble the matrix (36, 37, 38). The punctate FN staining observed in ADAMTS18 cultures might represent FN at its initial state, when compact FN dimers are bound to the cell surface through RGD integrins (36). However, because of ADAMTS18 cleavage of the N-terminal fragment, subsequent binding of other FNs is prevented, in contrast to control cultures where this can proceed to mature fibrils. ADAMTS18-truncated FN molecules, including N-terminal I_1_–_5_, are instead released into the medium, as was shown in the WBs. Despite the complexity of FN fibrillogenesis mechanisms, it is clear that N-terminal FN-I_1–5_ is important for the regulation of this process, and subsequently, ADAMTS18 cleavage of this fragment can have major consequences on the FN matrix.

The impact of ADAMTS18-mediated FN matrix regulation on essential endothelial processes was clearly evident in our studies. In particular, we observed that overexpression of ADAMTS18 reduced cell division and negatively affected cell proliferation. In the cell cycle analysis, we demonstrated that more cells were arrested in the G2/M phase and that fewer were in G0/G1. Consistently in ADAMTS18-overexpressing cells, we observed downregulation of the expression of many cell cycle–related genes, including *CDK1*, a master regulator of the cell cycle, particularly the transition of G2 to M phase (39). Furthermore, cell division relies on adhesion remodeling, which is governed in this phase of the cell cycle by integrin β1, which is the main receptor for FN (22). Dix *et al.* (40) showed that lack of FN coating, and therefore adhesion, arrested cell cycle (40). Of note, we also observed reduced expression of *ECT2*, which regulates the remodeling of the actomyosin cytoskeleton essential for mitotic cell rounding, which is associated with adhesion and allows cell division. Together, these results indicated that ADAMTS18, either directly through unknown mechanisms or indirectly through its modulation of FN matrix, prevented cells from completing their division to generate daughter cells. It is likely that it is ADAMTS18 prevention of FN fibrillogenesis that affected the cell cycle, since it is well known that adhesion to the ECM is essential for normal cell-cycle progression (41), subsequently remodeling, and stiffness of the ECM can significantly affect these processes (42). FN was previously reported to support EC survival (34), regulate EC morphology, behavior, and cell cycle (43), as well as induce EC migration (44, 45), and influence vascular morphogenesis (46, 47). Of note, FN coating was shown to significantly increase adhesion and proliferation of HUVECs compared with a noncoated surface (48, 49). ADAMTS18 modification of the FN matrix may also impact a cascade of other interactions and subsequently cellular processes; for example, in *Adamts18* knockout studies, the loss of ADAMTS18 resulted in abnormal FN accumulation, which dysregulated VEGF-A availability and subsequently affected EC behavior in both intestinal villi tips and liver sinusoids (14, 15).

To study the function of ADAMTS18 in EC, we also performed *ADAMTS18* knockdown by siRNA. Overall, the knockdown studies demonstrated a modest effect on HUVECs, as indicated by low FCs at the gene expression level and the absence of significant changes in functional assays. This may be technical in nature, as only 50% of the *ADAMTS18* transcripts were reduced; alternatively, it may be due to low albeit potentially specific expression of ADAMTS18 in EC. Nevertheless, the RNA-Seq studies indicated significant changes in adhesion-related genes, including *LAMA5* and *ITGB4*. LAMA5 is a component of the basement membrane and was found in EC basement membranes of mature vessels, predominantly in capillary ECs (50). Modulation of LAMA5 was also noted in *Adamts18* knockout mice (51, 52, 53). Importantly, LAMA5 was shown to interact with ADAMTS18, as an N-terminal fragment of LAMA5 coimmunoprecipitated with ADAMTS18 (54). In addition, we observed a decrease in ITGB4, a well-known integrin that binds LAMA5 (55). Integrins are heterodimeric receptors expressed on the cell membrane that serve a role as a structural link between cells and ECM and mediate signaling pathways. ITGB4 is expressed by blood vessels and ECs, including HUVECs (56), and was shown to regulate EC apoptosis and autophagy, to mediate senescence in HUVECs, and to be implicated in angiogenesis (57). Significantly, knockdown of *ITGB4* in HUVECs inhibited endothelial sprouting and migration *in vitro* (56), which correlates with our previous observations of reduced sprouting following *ADAMTS18* knockdown (13).

Of note, significant changes in gene expression with ADAMTS18 knockdown were already observed at 24 h, which may indicate yet unknown mechanisms of transcriptional regulation by ADAMTS18. Further studies of ADAMTS18 would be required to delineate this and other putative ADAMTS18 functions. For example, in this study, we identified a second site in FN that is proteolytically cleaved by ADAMTS18 at G^2288^–L^2289^. This site in FN was also previously demonstrated to be cleaved by ADAMTS9 (31). Interestingly, this cleavage truncates the FN molecule of the C-terminal domain that binds to fibrin (58, 59). Likewise, the N-terminal fragment FN-I_1–5_ generated by the other cleavage site contains the N-terminal fibrin-binding domain (60). However, the function of the C-terminal fibrin-binding domain is poorly understood, and further studies of this ADAMTS18-generated FN fragment would be necessary. These may also be facilitated by the generation of other ADAMTS18 mutants. Here, we generated a catalytic mutant of ADAMTS18, which was based on previous mutations described for other ADAMTSs (30, 31). Indeed, the mutation E437A does affect the catalytic activity of ADAMTS18, as indicated by mass spectrometry and WB data. However, data from the proliferation and cell cycle assessment demonstrated some effects of ADAMTS18-E437A mutant, which could indicate residual activity in the mutant ADAMTS18. Despite this, it was clear that ADAMTS18-E437A did not hinder FN assembly.

Overall, in this study, we demonstrated a molecular mechanism of ADAMTS18, which involves the cleavage of FN. We identified two cleavage sites of ADAMTS18 in FN and showed that this cleavage profoundly affects FN fibrillogenesis. Our results indicate that ADAMTS18 is a player in remodeling of the ECM in the endothelium, which may impact endothelial functions including adhesion and angiogenesis. These findings pave the way for a better understanding of ADAMTS18 function in biological processes and endothelial biology.

## Experimental procedures

### Cloning

For pLV-ADAMTS18 cloning, the human-ADAMTS18 sequence was amplified from pBluescriptR-hADAMTS18 plasmid using Phusion HF DNA Polymerase (Thermo Fisher Scientific) and the primers 5′-ACCGGTGCCACCATGGAGTGCGCCCTCC-3′ (AgeI site underlined) and 5′-GTCGACTCAGATCTTCCTTGTGCATGACTTG-3′ (SalI site underlined). The hADAMTS18 sequence was then cloned into a blunt cloning plasmid (pMini T 2.0; NEB PCR Cloning Kit; NEB), and its sequence was confirmed by Sanger sequencing (Macrogen Europe). By restriction digestion with AgeI and SalI restriction enzymes, pLV1-hPGK-GFP (pLV-GFP, (61)) LV plasmid was linearized, and the GFP sequence was replaced with the hADAMTS18 sequence from pMiniT2.0-hADAMTS18 to generate pLV-hPGK-hADAMTS18 (pLV-AD18).

The mutant pLV-hPGK-ADAMTS18-E437A (pLV-AD18-E437A) was obtained by subcloning a gBlocks gene fragment (IDTDNA) corresponding to ADAMTS18 nucleotide sequence 695 to 1476 with a point mutation A^1310^–C^1310^ into pLV-PGK-hADAMTS18. The original codon GAG has been mutated to GCG, leading to substitution of the glutamic acid in position 437 with alanine. The subcloning was performed by restriction digestion with NsiI and Bsu361.

For pLV-hPGK-stuffer DNA (pLV-SD) cloning, a 2709 bp SD sequence was similarly cloned into the AgeI and SalI sites in pLV1-hPGK-GFP replacing the GFP sequence with the SD sequence.

### Cell lines, cell culture, and transfections

Human cell lines, human embryonic kidney 293 (American Type Culture Collection [ATCC]; CRL-1573), 293T (ATCC; CRL-3216), EA.hy926 (ATCC; CRL-2922), and HPFc (pool of three donors, PromoCell), were cultured in Dulbecco’s modified Eagle’s medium-high glucose (Gibco) supplemented with 10% fetal bovine serum, 1% penicillin (100 mg/ml), and 1% streptomycin (100 U/ml). Primary HUVECs were isolated by collagenase digestion (62) from human umbilical cords obtained from the maternity ward of the Kuopio University Hospital, with the approval from the Research Ethics Committee of the Northern Savo Hospital District (Ethical permit no.: 143/2023, Research permit no.: 197/2023). HUVECs at early passage (passage 2–6) were maintained in endothelial cell growth medium (ECGM; PromoCell) supplemented with ECGM SupplementMix (PromoCell) as well as gentamicin (250 ng/ml) and amphotericin (10 μg/ml), in cell culture flasks coated with 10 μg/ml human FN (Corning; #354008) and 0.05% gelatin (Sigma). For RNA-Seq studies, a pool of three different donors was used for each transfection.

Cloned plasmids were transfected into 293T cells using Lipofectamine 3000 reagent (Thermo Fisher Scientific), according to the manufacturer’s instructions. For siRNA knockdown, dicer-substrate oligonucleotides hs.Ri.ADAMTS18.13.1, hs.Ri.ADAMTS18.13.2, hs.Ri.ADAMTS18.13.3 (sequences are, respectively, #5108262925′-rArCrArArCrUrUrGrArArArArGrUrArUrUrCrCrArArGrGAA-3′, #510826295 5′-rArGrGrUrGrArUrArArUrUrCrArArCrUrUrGrCrArArGrUTT-3′, and #510826298 5′-rCrArCrUrUrUrArUrUrGrUrCrCrArGrGrUrArCrUrUrGrGAA-3′), and DsiRNA control (TriEFCTA Kit DsiRNA Duples; IDTDNA) were transfected into ECs with a concentration of 10 nM using Oligofectamine (Thermo Fisher Scientific; #12252011) according to the manufacturer’s instruction. The cells were thereafter collected by trypsinization for downstream analysis at 24 h or 96 h (RNA expression by RT–qPCR, RNA-Seq, or WB) or experiments (adhesion, migration, and proliferation).

### Lentiviral production and transduction

Viral-like particles encapsulating LV-hPGK-hADAMTS18 or LV-hPGK-hADAMTS18-E437A or LV-hPGK-SD constructs were produced in 293T cells by cotransfection with the packaging constructs pVSV-g (63), pREV (64), and pMDg (65), using the calcium phosphate method as previously described (66). To perform transductions, cells were seeded at 50% confluence the day before transduction and the following day transduced with medium containing multiplicity of infection 10 of LV-ADAMTS18, LV-ADAMTS18-E437A, LV-SD, or LV-GFP viral vectors. The transducing medium was replaced with fresh culture medium 14 h after transduction and incubated for 1, 2, or 3 days (depending on the experiment) at 37 °C and 5% CO_2_. The cells were thereafter collected by trypsinization for downstream analysis (RNA expression by RT–qPCR, WB, or RNA-Seq) or experiments (proliferation, cell cycle analysis, FN fibrillogenesis, and mass spectrometry).

### RNA isolation and complementary DNA synthesis

For RNA-Seq, RNA was extracted from cells using the Qiagen RNeasy Plus Kit, according to the manufacturer’s instructions. For ddPCR analysis, RNA was extracted with the Qiagen RNeasy Kit according to the manufacturer's instructions. For other applications, total RNA isolation was performed using the TRIreagent method. Briefly, cells were lysed with TRIreagent, and RNA was separated from proteins and DNA by adding chloroform and phase separation. RNA was then precipitated with isopropanol, washed with ethanol, and resuspended in nuclease-free water. RNA was then subjected to DNase treatment with the TURBO DNA-free Kit (Thermo Fisher Scientific) followed by complementary DNA (cDNA) synthesis with RT using the RevertAid First Strand cDNA Synthesis Kit (Thermo Fisher Scientific).

### ddPCR and qPCR

For ADAMTS18 expression in different cell lines, mRNA expression was quantitated in cDNA samples using ddPCR, with 2× ddPCR Supermix for Probes (no dUTP; Bio-Rad) and specific primer TaqMan-based probe assays for *ADAMTS18* (HsPT581326160, IDTDNA) and *IPO8* (dHsaCPE5044719, Bio-Rad), according to the manufacturer's instructions. Droplets were generated with the QX200AutoDG Droplet Digital PCR System, and data were acquired with the QX200 DropletReader and analyzed with QX Manager Standard Edition (2.1.0). The ratio to the housekeeping gene *IPO8* is represented.

mRNA expression was quantitated in cDNA samples using real-time qPCR, with 2× Universal PCR Master Mix (Thermo Fisher Scientific), and specific primer TaqMan-based probe assays for *ADAMTS18* (Hs.PT.58.1326160, IDTDNA), *LAMA5* (Hs.PT.58.3171877, IDTDNA), *RBPJ* (Hs.PT.58.4105281, IDTDNA), *CXCL1* (Hs.PT.58.39039397, IDTDNA), *ITGB4* (Hs.PT.58.3714621, IDTDNA), *CDK1* (Hs.PT.58.3906949, IDTDNA), *MAD2L1* (Hs.PT.58.45564593, IDTDNA), *ECT2* (Hs.PT.58.26231644, IDTDNA), *FN1* (Hs.PT.58.40005963, IDTDNA), and *ACTB* (Hs.PT.39a.22214847, IDTDNA). Gene expression of interest was standardized to *ACTB* housekeeping gene, and the relative expression was calculated using the ΔΔCT method (67).

### RNA sequencing

Illumina-compatible sequencing libraries from the RNA samples were prepared at Azenta Life Sciences, who also performed the paired-end sequencing on Illumina NovaSeq (2 x 150). Sequence reads were trimmed to remove possible adapter sequences and nucleotides with poor quality using Trimmomatic, version 0.36. The trimmed reads were mapped to the *Homo sapiens* GRCh38 reference genome available on ENSEMBL using the STAR aligner, version 2.5.2b.

Bulk RNA-Seq raw gene-level counts were analyzed with the nf-core/differentialabundance pipeline, v1.5.0. A sample sheet mapped sample IDs to conditions, and a contrast file specified pairwise tests comprising siAD18 *versus* siNC at each time point for each replicate (replicates 1–3 for siAD18 with matched siNC controls), siAD18 96 h *versus* 24 h within replicate, and siNC 96 h *versus* 24 h. Differential expression was performed with DESeq2; gene-wise *p* values were adjusted for multiple testing using the Benjamini–Hochberg procedure to yield *p*_adj_ for each contrast. All analyses ran on the SLURM high-performance computing environment operated by the UEF Bioinformatics Center. For visualization, the pipeline’s variance-stabilizing transformation matrix was used to generate two heatmaps (24 h and 96 h) in R with pheatmap (version 1.0.13): at each time point, we subset predefined gene lists, centered, and scaled each gene across samples to row Z-scores, annotated columns by condition, clustered rows, and left columns unclustered.

### Western blot

Conditioned media and lysates were analyzed by WB. Protein content of the lysates was measured with the BCA kit (Pierce). Protein (10–30 μg) from lysates or 30 μl of conditioned media was subjected to SDS-PAGE on a 4% to 20% gel and electroblotted to polyvinylidene difluoride membranes. For detection of the N-terminal FN fragment, the conditioned media were concentrated by centrifugation at 3100 rpm for 5 min in Amicon Ultra 10,000 molecular weight cutoff (Millipore). After blocking in 5% bovine serum albumin in Tris-buffered saline with Tween-20, membranes were probed with polyclonal ITGB4 (sc-514426; Santa Cruz, 1:250 dilution), GAPDH (14C10; Cell Signaling Technology, 1:1000 dilution), hADAMTS18 (a kind gift from Dr Cathrin Brisken's lab [ISREC], 1:1000 dilution), polyclonal FN (#PA5-29578; Thermo Fisher Scientific, 1:2000 dilution), monoclonal N-terminal FN (#ab270240; Abcam, 1:500 dilution) primary antibodies. Horseradish peroxidase–conjugated anti-rabbit (#31460; Thermo Fisher Scientific) or anti-mouse (#115-035-003; Jackson ImmunoResearch) secondary antibodies were detected with ECL substrate (Pierce ECL Plus Western Blotting Substrate; ThermoScientific) and imaged with a ChemiDoc XRS (Bio-Rad). Protein intensity was quantitated in Image Lab Software 6.0 as the adjusted volume (Int) of the bands. The intensity of the protein of interest was normalized to GAPDH, and then the obtained values were normalized to the control.

### Cell adhesion, cell migration, cell proliferation, and cell cycle analysis

Cell adhesion, migration, and proliferation were evaluated with xCELLigence Real-Time Cell Analyzer Multiplate (RTCA MP) instrument (ACEA Biosciences), according to the manufacturer's instructions.

For cell proliferation, HUVECs were seeded in E-plate 16 PET (ACEA Biosciences) at a density of 5 × 10^3^ per well 24 h after transfection with DsiRNA or transduction with LVs. Prior to seeding, wells were coated with 10 μg/ml FN (Corning; #354008) and 0.05% gelatin (Sigma). The plate was incubated in RTCA MP at 37 °C and 5% CO_2_. Cell proliferation was monitored every 2 h for 80 h. For cell adhesion, HUVECs were seeded in E-plate 16 PET (ACEA Biosciences) at a density of 1 × 10^4^ per well 96 h after transfection with DsiRNA or 72 h after transduction with LVs. Prior to seeding, wells were coated with 10 μg/ml FN and 0.05% gelatin, and the plate was incubated in RTCA MP at 37 °C and 5% CO_2_. Cell adhesion was monitored every 15 min for 2 h.

For cell migration, HUVECs were seeded in a CIM plate 16 (ACEA Biosciences) according to the manufacturer's instructions. Briefly, the bottom chamber of a CIM plate 16 was filled with complete ECGM supplemented with VEGF-A 50 ng/ml as a chemoattractant. HUVECs transfected with DsiRNA were seeded 96 h after transfection in the upper chamber of the CIM plate 16 at a density of 2 × 10^4^ per well in serum-free medium. Prior seeding, the bottom surface of the wells of the upper chamber was coated with 10 μg/ml FN and 0.05% gelatin. The plate was incubated in RTCA MP at 37 °C and 5% CO_2_. Cell migration was monitored every 15 min for 24 h.

Cell cycle analysis was performed using NucleoCounter NC3000 (Chemometec), Two-Step Cell Cycle Assay, according to the manufacturer’s instructions. HUVECs were transduced with LV-AD18, LV-AD18-E437A, and LV-SD in duplicate wells of a 6-well plate. Seventy-two hours after transduction, cell lysates were treated according to the manufacturer’s instructions, loaded on an NC slide A2 (Chemometec), and analyzed in NucleoCounter NC3000 using the Two-Step Cell Cycle Assay program.

### Immunofluorescence

After transduction of HUVECs with LV-AD18, LV-AD18-E437A, and LV-SD for 48 h, cells were seeded in chamber slides coated with 30 μg/ml human FN (Corning; #354008) at a density of 5 × 10^4^ per well. Cells were maintained for 24 h and 48 h in complete ECGM and then fixed with 4% paraformaldehyde in PBS (w/v).

Alternatively, after transduction of HUVECs with LV-AD18, LV-AD18-E437A, and LV-SD for 48 h, cells were seeded together with HPFc on chamber slides coated with 0.5% gelatin at a ratio of 10:1 and a total density of 1.75 × 10^4^ per well. Cells were maintained for 6 days in complete ECGM and then fixed with 4% paraformaldehyde in PBS (w/v).

After fixation, cells were permeabilized, blocked with 2% bovine serum albumin in Triton X-100, 1% PBS, and incubated with anti-FN polyclonal antibody (#PA5-29578; Thermo Fisher Scientific) at a dilution of 1:200, followed by incubation with Alexa Fluor 488 goat anti-rabbit (#A11008; Thermo Fisher Scientific)–conjugated secondary antibody. The slides were mounted with VECTASHIELD Antifade Mounting Medium with 4′,6-diamidino-2-phenylindole (H1200; Vector Laboratories) and imaged with a Nikon Eclipse NIE microscope and Nikon DS-Ri2 camera, with objectives Plan Fluor 10×/0.30 and Plan Fluor 20×/0.50. Identical camera settings were used for comparing different groups, and images were analyzed using ImageJ, version 2.9.0 software (US National Institutes of Health). Nuclei count was quantified with ImageJ. Quantification was performed with ImageJ (brightness adjusted to 45–248, threshold adjusted at 0–220), and mean intensity was plotted in a graph for comparison (n = 9 images).

### Proteomic determination of ADAMTS18 cleavage of FN

For determination of ADAMTS18 cleavage of FN, HUVECs were transduced with LV-AD18, LV-AD18-E437A, and LV-SD, and 48 h after transduction, cells were seeded 5 × 10^4^ per well in a 6-well plate precoated with 30 μg/ml human FN in complete ECGM (three wells for each group). Cells were allowed to adhere, and 3 h after seeding, the complete medium was replaced with serum-free ECGM. Cells were cultured for 24 h, and then media and lysates were collected for analysis. For proteomic analysis, conditioned media from the same groups were pooled together, and proteins in the media were precipitated with trichloroacetic acid. Pellets were washed with HPLC-grade ice-cold acetone and resuspended in 0.5 M Tris (pH 8.6) containing 6 M guanidine hydrochloride. Following reduction with TCEP and alkylation with iodoacetamide, proteins were dissolved in 1.2 M urea in 0.1 M Tris–HCl (pH 8.5). Proteins were digested with LysC (Promega, rLys-C, Mass Spec Grade #V167A) and TPCK–trypsin (Promega, sequencing grade modified trypsin #V511C). The samples were dissolved in 50 μl of 2% acetonitrile acidified with 10 μl of 0.1% formic acid and centrifuged for mass spectrometry experiments prior to LC–MS/MS analysis.

### LC–MS/MS protein analysis

The mass spectrometric analysis was performed with a Q-Exactive Focus Orbitrap mass spectrometer (Thermo Scientific) coupled with a Vanquish UPLC system (Thermo Scientific) with data-dependent acquisition mode. Chromatographic separation was performed using an Advance Bio Peptide Map column (2.1 × 250 mm; 2.7 μm). A 90 min gradient elution with (A) 0.1% formic acid in water and (B) 0.1% formic acid in acetonitrile was used. The gradient elution was as follows: 0 to 2 min 2 → 7% of B, 2 to 5 min 7%, 5 to 50 min 7 → 30%, 50 to 83 min 30 → 45%, 83 to 83.5 min 45 → 80%, 83.5 to 85.5 80%, 85.5 to 86 min 80 → 2%, and 86 to 90 min equilibrium at 2%. A constant flow rate of 0.3 ml/min and a constant column oven temperature of 40 °C was used. The injection volume was 20 μl (n = 3 injections). Data-dependent acquisition was performed in full scan positive mode, scanning from 375 to 1500 *m/z*, with an MS1 resolution of 70,000, an automatic gain control target of 3 × 10^6,^ and a maximum inject time of 100 ms. The top 10 most intense ions from each MS1 scan were selected for collision-induced dissociation. MS2 resolution was set at 35,000 with the automatic gain control target of 2 × 10^5^ and maximum inject time of 114 ms with an isolation window of 2 *m/z*, scanning range of 200 to 2000 *m/z,* and normalized collision energy of 28.

The quantification of proteins and the discovery of novel cleavage sites of FN were done with Skyline software, version 24.1.0.199. A protein sequence database of reviewed *H. sapiens* (UP000005640) from UniProt was used, as well as FN sequence with code P02751 from UniProt (Table S3). Oxidation of methionine, acetylation of protein-N termini, deamidation at asparagine and glutamine, oxidation at methionine, and phosphorylation at serine/threonine were set as variable modifications. Carbamidomethylation of cysteine was set as a fixed modification. Specific digestion mode was used for Trypsin/P, LysC/P, and variable proteases to discover novel cleavage sites of FN (Table S4). The mass accuracy was set to 20 ppm, two precursor charges were set, and maximum missed cleavages were set as two. The MSAmanda search engine was used with MS1 and MS2 tolerances of 5 and 10 ppm, respectively. The maximum variable modifications were set as three.

### Statistical analysis

Data were statistically analyzed using GraphPad Prism 10.6.1 (GraphPad Software, Inc). The data are presented as mean ± SD, n represents the number of independent experiments unless otherwise specified. Statistical significance was ascribed at *p* < 0.05, using two-tailed analysis. Data that demonstrated homogeneous variance were tested using Student’s *t* test or multiple ANOVA and including lognormal ordinary one-way ANOVA for log-transformed data, and Tukey’s multiple comparison; otherwise, nonparametric data were tested using Kruskal–Wallis analysis with Dunn’s multiple comparison for three or more groups. All numerical data were analyzed statistically, but only significant differences are represented in the graphs. For RNA-Seq data, gene-wise *p* values were adjusted for multiple testing using the Benjamini–Hochberg procedure to yield *p*_adj_ for each contrast.

## Data availability

RNA-Seq data obtained in this study are available from the National Center for Biotechnology Information Gene Expression Omnibus portal (accession numbers: GSE278198 and GSE306600). Mass spectrometry data are available from the ProteomeXchange Consortium database, permanent link https://panoramaweb.org/rnqlE0.url and ProteomeXchange ID PXD060097.

## Supporting information

This article contains supporting information (Tables S1–S4, Fig. S1).

## Conflict of interest

The authors declare that they have no conflicts of interest with the contents of this article.
