# Supplementary material for: A disintegrin and metalloproteinase with thrombospondin motifs 18 (ADAMTS18) cleaves fibronectin and negatively regulates its fibrillogenesis
Source: J Biol Chem. 2025 Oct 22;301(12):110844. doi: 10.1016/j.jbc.2025.110844 (PMC12666562; doi:10.1016/j.jbc.2025.110844)
Supplement: Supporting information [file mmc1.pdf]

## SUPPORTING INFORMATION

# *A disintegrin and metalloproteinase with thrombospondin motifs 18 (ADAMTS18) cleaves fibronectin and negatively regulates its fibrillogenesis*

Maria Barbiera[1], Mikko Gynther[2], Tetsuya Terasaki[2], Suvi Sauhiainen[1], Johanna P. Laakkonen[1], Michael Jeltsch[3], Seppo Ylä-Herttuala[1,4], Nihay Laham-Karam[1,*]

**Supporting Table S1.** Table of significant differentially expressed genes (padj<0.5) identified in all the three siRNAs targeting ADAMTS18 24h after transfection of HUVECs.

| | **siAD18-1** | | **siAD18-2** | | **siAD18_3** | |
|---|---|---|---|---|---|---|
| Gene_name | log2FC | padj | log2FC | padj | log2FC | padj |
| *CD164* | 0.10 | 1.59E-03 | -0.45 | 4.49E-45 | 0.08 | 3.01E-02 |
| *HMGA2* | -0.11 | 6.25E-03 | -0.40 | 1.10E-22 | 0.11 | 8.30E-03 |
| *CAV1* | -0.33 | 5.37E-58 | -0.17 | 1.87E-17 | 0.11 | 2.99E-08 |
| *SLC2A3* | 0.13 | 4.49E-02 | 0.72 | 3.10E-17 | -0.32 | 6.94E-04 |
| *RBPJ* | -0.41 | 1.83E-09 | -0.54 | 1.44E-16 | -0.38 | 2.00E-08 |
| *ADAMTS18* | -0.57 | 1.39E-07 | -0.96 | 6.81E-16 | -0.83 | 3.16E-11 |
| *COL27A1* | 0.18 | 1.40E-02 | 0.60 | 4.34E-14 | 0.17 | 1.69E-02 |
| *ARMCX2* | -0.10 | 3.03E-02 | -0.33 | 1.56E-12 | -0.17 | 4.09E-04 |
| *TGFBR2* | -0.45 | 6.15E-34 | 0.26 | 4.82E-12 | 0.25 | 2.91E-11 |
| *GBP1* | 0.18 | 2.42E-03 | 0.41 | 1.12E-11 | 0.55 | 3.31E-19 |
| *GBA2* | 0.20 | 1.18E-06 | 0.26 | 1.29E-11 | -0.31 | 2.18E-14 |
| *S1PR1* | 0.17 | 1.04E-06 | 0.21 | 8.40E-10 | 0.15 | 7.36E-06 |
| *FHL2* | -0.09 | 3.78E-02 | -0.26 | 2.50E-09 | -0.10 | 2.62E-02 |
| *C2CD5* | 0.21 | 3.96E-04 | 0.31 | 9.44E-08 | 0.16 | 5.07E-03 |
| *ERLIN1* | -0.34 | 1.91E-17 | 0.18 | 1.14E-06 | 0.11 | 6.40E-03 |
| *MAP2K3* | 0.14 | 1.02E-02 | 0.26 | 2.64E-06 | 0.11 | 4.52E-02 |
| *H1F0* | -0.12 | 8.48E-05 | -0.15 | 3.29E-06 | 0.10 | 1.12E-03 |
| *GLMP* | 0.10 | 4.03E-02 | -0.24 | 6.02E-06 | 0.10 | 4.15E-02 |
| *PLXNA1* | 0.20 | 1.37E-03 | 0.28 | 6.37E-06 | 0.20 | 1.38E-03 |
| *KIT* | -0.20 | 2.08E-03 | -0.30 | 8.01E-06 | -0.51 | 7.82E-13 |
| *TMEM87B* | 0.10 | 4.60E-02 | 0.26 | 1.40E-05 | 0.16 | 6.26E-03 |
| *NBEAL2* | 0.24 | 8.80E-05 | 0.25 | 2.81E-05 | 0.22 | 2.88E-04 |
| *HDGFL3* | -0.11 | 5.45E-04 | -0.13 | 3.22E-05 | -0.21 | 1.04E-10 |
| *CLN8* | 0.13 | 1.33E-02 | -0.22 | 7.53E-05 | -0.52 | 1.27E-17 |
| *SZRD1* | -0.18 | 6.15E-07 | -0.14 | 1.11E-04 | -0.11 | 2.78E-03 |
| *LAMA5* | 0.34 | 2.63E-04 | 0.31 | 2.08E-04 | 0.20 | 9.26E-03 |
| *TUBGCP6* | 0.34 | 1.27E-05 | 0.26 | 2.27E-04 | 0.13 | 3.62E-02 |
| *ARMCX1* | 0.25 | 4.70E-04 | 0.25 | 2.52E-04 | 0.21 | 2.14E-03 |
| *FGD4* | -0.15 | 9.28E-03 | -0.22 | 2.88E-04 | -0.19 | 2.39E-03 |
| *TEAD2* | 0.08 | 4.07E-02 | 0.14 | 3.00E-04 | -0.15 | 3.67E-04 |
| *MZT1* | -0.44 | 4.23E-20 | -0.16 | 5.87E-04 | -0.51 | 5.73E-25 |
| *MYO1E* | -0.28 | 1.60E-13 | 0.12 | 6.00E-04 | -0.10 | 7.75E-03 |
| *PCTP* | 0.17 | 1.02E-02 | 0.23 | 7.50E-04 | -0.20 | 5.33E-03 |
| *FDPS* | -0.08 | 4.79E-02 | -0.13 | 7.59E-04 | -0.13 | 5.31E-04 |
| *RRN3P1* | 0.26 | 9.28E-03 | 0.39 | 8.10E-04 | 0.17 | 4.46E-02 |
| *JRK* | 0.12 | 3.70E-02 | 0.20 | 1.37E-03 | 0.21 | 1.25E-03 |

| | | | | | | |
|---|---|---|---|---|---|---|
| *PLAT* | -0.21 | 6.11E-05 | 0.15 | 1.94E-03 | -0.25 | 1.68E-06 |
| *SLC1A1* | -0.17 | 6.63E-03 | -0.20 | 2.15E-03 | 0.12 | 3.26E-02 |
| *XYLT2* | 0.19 | 4.04E-04 | 0.15 | 3.01E-03 | 0.11 | 3.35E-02 |
| *DVL3* | 0.12 | 1.08E-02 | 0.14 | 3.59E-03 | 0.13 | 6.84E-03 |
| *DHX15* | -0.09 | 5.66E-03 | 0.09 | 4.21E-03 | -0.08 | 1.69E-02 |
| *ERAP1* | 0.09 | 3.85E-02 | -0.12 | 4.65E-03 | 0.24 | 1.54E-08 |
| *MT-ND3* | 0.17 | 2.16E-02 | 0.22 | 5.22E-03 | -0.21 | 9.06E-03 |
| *EFNA1* | 0.21 | 1.51E-03 | 0.17 | 7.39E-03 | -0.36 | 5.34E-07 |
| *CFL2* | -0.19 | 6.20E-05 | -0.12 | 9.48E-03 | -0.10 | 2.46E-02 |
| *CDC42SE2* | -0.11 | 3.95E-02 | -0.15 | 1.10E-02 | -0.17 | 4.30E-03 |
| *GNG12* | -0.09 | 3.46E-02 | -0.11 | 1.18E-02 | -0.14 | 8.95E-04 |
| *DHX58* | 0.67 | 8.49E-07 | 0.24 | 1.25E-02 | 1.70 | 3.24E-19 |
| *SSR1* | -0.12 | 3.51E-04 | -0.09 | 1.44E-02 | -0.09 | 1.23E-02 |
| *UBE2D3* | -0.15 | 8.94E-08 | -0.07 | 1.51E-02 | -0.08 | 4.74E-03 |
| *TMSB4X* | -0.09 | 2.16E-02 | -0.09 | 1.71E-02 | -0.08 | 4.17E-02 |
| *LIMK1* | -0.35 | 3.85E-08 | 0.13 | 1.80E-02 | -0.18 | 2.06E-03 |
| *BTN3A2* | 0.13 | 4.07E-02 | 0.17 | 1.83E-02 | 0.45 | 2.69E-07 |
| *PLEKHB2* | 0.12 | 9.60E-04 | 0.09 | 1.95E-02 | -0.11 | 5.54E-03 |
| *LAMP1* | 0.07 | 3.22E-02 | -0.08 | 2.02E-02 | 0.08 | 1.02E-02 |
| *TLR4* | -0.30 | 2.34E-08 | -0.12 | 2.20E-02 | -0.42 | 2.28E-15 |
| *GMPR* | 0.19 | 7.44E-03 | -0.15 | 2.34E-02 | 0.43 | 2.93E-08 |
| *STRAP* | -0.07 | 4.96E-02 | -0.08 | 2.54E-02 | -0.12 | 5.78E-04 |
| *BMP6* | 0.15 | 3.01E-08 | -0.07 | 2.55E-02 | 0.12 | 1.11E-05 |
| *PRICKLE1* | -0.18 | 1.37E-03 | -0.12 | 2.55E-02 | 0.14 | 9.40E-03 |
| *TET3* | -0.13 | 4.03E-02 | 0.15 | 2.72E-02 | 0.13 | 4.69E-02 |
| *ADAMTS1* | 0.33 | 2.03E-05 | 0.14 | 3.01E-02 | 0.58 | 9.71E-15 |
| *SRSF1* | -0.09 | 8.53E-03 | 0.07 | 4.27E-02 | -0.08 | 2.04E-02 |
| *FAM98A* | -0.14 | 5.61E-06 | 0.07 | 4.40E-02 | -0.07 | 4.02E-02 |
| *MYO6* | -0.10 | 1.54E-02 | -0.08 | 4.42E-02 | -0.09 | 1.99E-02 |
| *HNRNPA0* | -0.08 | 4.59E-02 | -0.08 | 4.60E-02 | -0.17 | 7.70E-06 |
| *AGRN* | 0.14 | 4.77E-03 | 0.10 | 4.68E-02 | 0.28 | 4.39E-08 |
| *SLC25A13* | 0.21 | 3.66E-04 | 0.11 | 4.71E-02 | 0.19 | 8.58E-04 |
| *PPM1F* | -0.63 | 3.76E-48 | 0.09 | 4.74E-02 | -0.21 | 2.21E-06 |

**Supporting Table S2.** Table of significant differentially expressed genes (padj<0.5) identified in all the three siRNAs targeting ADAMTS18 96h after transfection of HUVECs.

| | **siAD18-1** | | **siAD18-2** | | **siAD18_3** | |
|---|---|---|---|---|---|---|
| Gene_name | log2FC | padj | log2FC | padj | log2FC | padj |
| *ADAMTS18* | -1.24 | 2.18E-22 | -0.55 | 2.34E-07 | -0.76 | 1.00E-09 |
| *CDH13* | -0.28 | 1.27E-17 | 0.23 | 3.78E-12 | 0.11 | 5.89E-04 |
| *COL18A1* | -0.28 | 2.54E-13 | 0.23 | 4.22E-09 | -0.12 | 1.18E-03 |
| *HSPD1* | 0.22 | 1.15E-11 | -0.10 | 3.03E-03 | 0.08 | 2.38E-02 |
| *IGF2* | -0.35 | 1.28E-11 | 0.26 | 1.18E-06 | -0.33 | 5.36E-10 |
| *NID1* | -0.33 | 2.95E-10 | 0.20 | 2.23E-04 | -0.13 | 7.15E-03 |
| *IL1RL1* | 0.31 | 1.12E-09 | -0.21 | 1.25E-04 | 0.33 | 1.48E-09 |
| *RAN* | 0.19 | 5.96E-09 | -0.10 | 3.12E-03 | 0.09 | 9.76E-03 |
| *EPAS1* | -0.20 | 7.96E-08 | 0.09 | 2.79E-02 | -0.20 | 7.70E-08 |
| *COL4A2* | -0.17 | 4.09E-07 | 0.23 | 1.83E-12 | -0.14 | 1.25E-05 |
| *MAGI1* | -0.26 | 5.82E-07 | 0.10 | 3.65E-02 | -0.11 | 2.48E-02 |
| *TNFSF4* | 0.40 | 9.53E-07 | 0.19 | 1.51E-02 | 0.88 | 4.45E-19 |
| *HSPA8* | 0.15 | 1.04E-06 | -0.12 | 7.38E-05 | 0.08 | 1.60E-02 |
| *CXCL1* | 0.51 | 2.24E-06 | 0.20 | 2.06E-02 | 0.79 | 5.24E-09 |
| *PSMD14* | 0.20 | 2.59E-06 | -0.10 | 1.73E-02 | 0.10 | 1.75E-02 |
| *RCN2* | 0.22 | 4.20E-06 | 0.12 | 1.52E-02 | 0.09 | 4.60E-02 |
| *EIF4G2* | 0.09 | 5.34E-06 | -0.06 | 9.16E-03 | 0.05 | 4.12E-02 |
| *PAICS* | 0.19 | 7.20E-06 | -0.11 | 7.57E-03 | 0.09 | 2.45E-02 |
| *CSE1L* | 0.19 | 7.26E-06 | -0.12 | 5.85E-03 | 0.09 | 3.84E-02 |
| *TGFBR2* | -0.16 | 1.54E-05 | 0.24 | 5.10E-10 | 0.08 | 4.59E-02 |
| *ITGB4* | -0.31 | 1.56E-05 | -0.44 | 1.43E-07 | -0.45 | 2.27E-07 |
| *FLT4* | -0.22 | 1.78E-05 | 0.28 | 3.81E-07 | -0.34 | 5.43E-10 |
| *LAMB2* | -0.14 | 5.96E-05 | 0.09 | 8.66E-03 | -0.11 | 1.08E-03 |
| *LTBP4* | -0.20 | 1.55E-04 | 0.15 | 4.72E-03 | -0.32 | 1.00E-07 |
| *CD164* | 0.11 | 1.20E-03 | -0.10 | 4.71E-03 | 0.08 | 3.52E-02 |
| *TCIM* | 0.21 | 1.21E-03 | 0.44 | 9.07E-08 | 0.24 | 1.67E-03 |
| *TNS1* | -0.18 | 1.36E-03 | 0.14 | 9.98E-03 | -0.19 | 1.11E-03 |
| *WDR6* | -0.12 | 1.86E-03 | -0.09 | 1.04E-02 | -0.09 | 1.99E-02 |
| *AEBP1* | -0.14 | 2.20E-03 | -0.10 | 2.19E-02 | -0.10 | 2.70E-02 |
| *ITGB3* | 0.12 | 2.52E-03 | 0.10 | 1.06E-02 | 0.16 | 1.93E-05 |
| *CLDND1* | 0.14 | 2.67E-03 | 0.09 | 3.79E-02 | 0.09 | 4.90E-02 |
| *PPP1R18* | -0.11 | 4.18E-03 | -0.11 | 3.46E-03 | -0.08 | 2.87E-02 |
| *DDIT4* | -0.16 | 5.22E-03 | 0.14 | 1.12E-02 | -0.22 | 3.28E-04 |
| *ABCG2* | -0.18 | 8.59E-03 | 0.19 | 1.86E-02 | -0.28 | 2.29E-03 |
| *YWHAE* | 0.08 | 8.67E-03 | -0.07 | 3.45E-02 | 0.07 | 1.75E-02 |
| *TJP2* | -0.09 | 1.22E-02 | -0.09 | 9.26E-03 | -0.11 | 9.27E-04 |

| | | | | | | |
|---|---|---|---|---|---|---|
| *CELSR1* | -0.15 | 1.27E-02 | 0.27 | 3.30E-04 | -0.26 | 4.37E-04 |
| *LGALS1* | 0.13 | 1.42E-02 | -0.13 | 1.02E-02 | 0.12 | 9.58E-03 |
| *SPTBN2* | -0.13 | 1.76E-02 | -0.11 | 4.09E-02 | -0.12 | 1.75E-02 |
| *ADD1* | -0.07 | 2.04E-02 | -0.08 | 6.66E-03 | -0.06 | 3.78E-02 |
| *MDM2* | 0.14 | 2.17E-02 | 0.12 | 4.37E-02 | 0.16 | 8.43E-03 |
| *HMGA2* | 0.10 | 2.47E-02 | -0.24 | 3.99E-07 | 0.24 | 2.80E-07 |
| *SPOCD1* | 0.13 | 2.58E-02 | 0.36 | 7.37E-08 | 0.29 | 2.04E-05 |
| *GATA2* | -0.10 | 3.03E-02 | -0.18 | 4.82E-05 | -0.10 | 2.67E-02 |
| *GBP1* | 0.13 | 3.28E-02 | 0.33 | 2.01E-06 | 0.13 | 1.36E-02 |
| *HINT1* | 0.10 | 3.71E-02 | -0.09 | 3.15E-02 | 0.11 | 9.65E-03 |
| *NYNRIN* | -0.10 | 3.80E-02 | -0.10 | 3.47E-02 | -0.11 | 8.50E-03 |
| *CTHRC1* | 0.11 | 3.95E-02 | 0.13 | 5.93E-03 | 0.12 | 9.46E-03 |
| *TIMP2* | -0.08 | 4.76E-02 | -0.10 | 1.12E-02 | -0.09 | 2.91E-02 |

**Supporting Figure S1.**

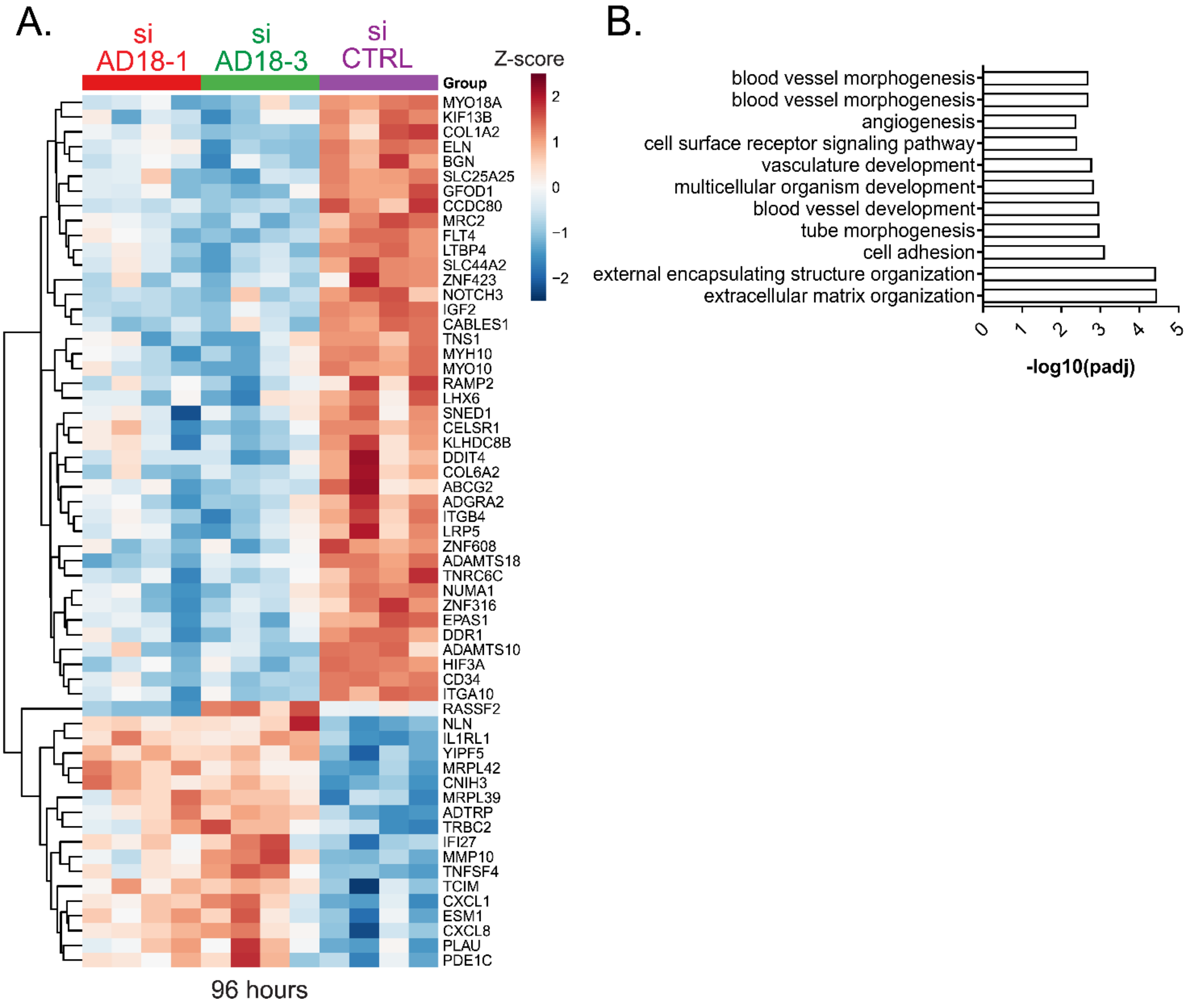


**Figure S1. Effect of *ADAMTS18* knockdown with siAD18-1 and siAD18-3 on gene expression.** ***A***) Heatmap representing significant DEGs (padj<0.05 and log2FC>0.15 or <-0.15) common to siAD18-1 and siAD18-3 (excluding siAD18-2) at 96h after siRNA transfection. ***B***) GO analysis (g:Profiler) of significant DEGs common to siAD18-1 and siAD18-3 at 24h and at 96h after transfection.

**Supporting Table S3.** Details of proteins identified by Mass Spectrometry

| Protein Name | Protein Accession | Protein Gene | Protein Sequence Coverage | molecular weight (Da) | Number of unique sequence |
|---|---|---|---|---|---|
| sp\|P02751\|FINC_HUMAN | P02751 | FN1 | 44 % | 272320 | 77 |
| sp\|Q8TE60\|ATS18_HUMAN | Q8TE60 | ADAMTS18 | 10 % | 135167 | 8 |

**Supporting Table S4.** Details of peptides identified by Mass Spectrometry

| Peptide sequence | observed mass (m/z) | post-translational modifications |
|---|---|---|
| $^{291}$AAVYQPQPHPQPPPYGHCVTDSGVVYSVGMQWLK$^{324}$ | 1265.28 | Carbamidomethyl (C) |
| $^{293}$VYQPQPHPQPPPYGHCVTDSGVVYSVGMQWLK$^{324}$ | 1217.921903 | Carbamidomethyl (C) |
| $^{2289}$LNQPTDDSCFDPYTVSHYAVGDEWER$^{2314}$ | 1034.445 | Carbamidomethyl (C) |
